# Wolf–Hunting Dog Interactions in a Biodiversity Hot Spot Area in Northern Greece: Preliminary Assessment and Implications for Conservation in the Dadia-Lefkimi-Soufli Forest National Park and Adjacent Areas

**DOI:** 10.3390/ani11113235

**Published:** 2021-11-12

**Authors:** Yorgos Iliopoulos, Eirini Antoniadi, Elzbieta Kret, Sylvia Zakkak, Theodora Skartsi

**Affiliations:** 1 WWF Greece, Lempesi 21, GR-117 43 Athens, Greece; e.kret@wwf.gr (E.K.); d.skartsi@wwf.gr (T.S.); 2 Callisto Wildlife Society, Mitropoleos 123, GR-54621 Thessaloniki, Greece; eiriniant@biol.uoa.gr; 3 Department of Biological Applications and Technologies, University of Ioannina Campus, University of Ioannina, Stavros Niarchos Avenue, GR-45110 Ioannina, Greece; 4 Department of Biology, National and Kapodistrian University of Athens, Panepistimioupoli, Ilisia, GR-15701 Athens, Greece; 5 Management Body of Dadia-Lefkimi-Soufli Forest National Park, GR-68400 Dadia, Greece; zak.sylvia@gmail.com

**Keywords:** *Canis lupus*, hunting dogs, depredation, prey selection, predation risk maps, Dadia Forest National Park, wildlife poisoning

## Abstract

**Simple Summary:**

Wolf attacks on hunting dogs are on the rise in many European countries, triggering retaliatory killing and poisoning of wolves. Poisoning may have detrimental effects on endangered vulture species. In critical areas for vulture conservation such as the Dadia-Lefkimi-Soufli Forest National Park, the conflict should be urgently evaluated. We assessed levels, trends, and defined related factors, by interviewing hunters and undertaking a diet analysis of wolf scats. Attacks affected mostly hare hunters, certain dog breeds and age classes, averaged one dog per hunter and decade, and happened under certain circumstances. Affected areas had specific landscape characteristics, fewer livestock, more hunting, and presence of wolf reproduction. Trends of wolf attacks on hunting dogs were positive and those on livestock negative. Wolves fed mainly on roe deer in summer and wild boar in winter, while the presence of dogs in scats was 5.1% in winter. Reduced dependence of wolves on livestock, as well as changes in wolf diet and hunting practices, may have predisposed wolves to kill more dogs recently. Wild boar also injured or killed hunting dogs, very often perplexing assessment of the conflict. The study concludes on practical measures for verifying and reducing hunting dog losses from wolf attacks.

**Abstract:**

Hunting dog depredation by wolves triggers retaliatory killing, with negative impacts on wildlife conservation. In the wider area of the Dadia-Lefkimi-Soufli Forest National Park, reports on such incidents have increased lately. To investigate this conflict, we interviewed 56 affected hunters, conducted wolf trophic analysis, analyzed trends for 2010–2020, applied MAXENT models for risk-map creation, and GLMs to explore factors related to depredation levels. Losses averaged approximately one dog per decade and hunter showing a positive trend, while livestock depredations showed a negative trend. Wolves preyed mainly on wild prey, with dogs consisting of 5.1% of the winter diet. Low altitude areas, with low to medium livestock availability favoring wolf prey and game species, were the riskiest. Dogs were more vulnerable during hare hunting and attacks more frequent during wolf post-weaning season or in wolf territories with reproduction. Hunter experience and group hunting reduced losses. Wolves avoided larger breeds or older dogs. Making noise or closely keeping dogs reduced attack severity. Protective dog vests, risk maps, and enhancing wolf natural prey availability are further measures to be considered, along with a proper verification system to confirm and effectively separate wolf attacks from wild boar attacks, which were also common.

## 1. Introduction

Dog predation by wildlife is one of the least studied interactions with wolves, the most common predator [1]. Wolves killing dogs is not a new phenomenon. Published records start in the 1980s, from several countries such as Italy [2], Belarus [3], the Iberian Peninsula [4,5,6], Finland [7,8], India [9], and North America [10,11,12]. Wolves also prey on a variety of other mesopredators, but dogs are the most commonly killed carnivores [13].

Wolf predation on dogs may be linked to the recovery of previously extinct wolf populations [11], or shortage of wild prey that forces wolves to switch their diet [3]. Wolves may kill dogs during game hunting [14]; in back yards [7]; when defending their territories [15]; and may, in all cases, extensively consume them afterwards [10,16,17,18]. Dog biomass in wolf diet varies considerably and up to 20%, although in most cases, it is below 5% [13]. Predation on dogs may involve only a small proportion of wolf packs in a population [19], with specific packs specialized in dog killing [7]. Dog availability in a wolf habitat constitutes an important factor shaping wolf–dog interactions [1].

Wolves kill all sorts of dogs, from strays, feral, and livestock guarding dogs (LGDs) to hunting dogs. The percentage of hunting dog fatalities varies from 30% in Finland [16] and up to 87% in Wisconsin, United States [11]. Human tolerance of dog depredations is strongly related to the type of dogs killed, with feral dog killing not triggering negative reaction, or even considered as an ecological service provided by wolves, as free-ranging dogs, when numerous, cause considerable damage to wildlife, or transmit diseases [20]. However, when dogs killed are pet or hunting dogs, a strong negative reaction and emotions are initiated against wolves [1].

In Greece, wolf depredation on dogs has been common in the past and mainly involved stray dogs. However, only recently, hunters have been claiming many incidents of wolf–hunting dog interactions (i.e., 321 cases in northern Greece, from 2014 to 2018 [21]). Contextualization of the wolf–hunting dog interactions in Greece during the last decade has been facilitated by the integration of GPS hunting dog collars. Tracking hunting dogs enables a fast retrieval of killed dogs, unlike in the past.

Hunting dogs are considered family members [22] and wolf depredation on those dogs greatly weakens tolerance to wolves by local societies [23,24]. Therefore, retaliatory killing of wolves, by any available means, may be the result of poor management of the wolf–hunting dog conflict. In a relevant analogous example, instances of dingoes predating on hunting dogs has reinstated poison use in Australia [1].

Wildlife poisoning threatens the sustainable management of ecosystems and biodiversity conservation worldwide [25]. The use of poison against wildlife has been banned throughout Europe since the end of 1980s/beginning of 1990s, with the ratification of the Bern Convention. However, in many areas, it remains a widely used illegal practice, mainly among livestock breeders and hunters [26,27,28]. While in most cases wolves or other carnivores are being targeted, many species of birds of prey become victims of unintentional poisoning [28,29]. Poisoning has been identified as one of the major threats to vulture populations in the Balkans. Approximately 100 cases involving ca. 200 vultures have been documented from 2008 to 2017, in five countries [30,31,32,33,34]. The Dadia-Lefkimi-Soufli Forest National Park (hereafter, DNP) in northern Greece is the home of the only Cinereous vulture (*Aegypius monachus*) breeding colony in the Balkans [35], and hosts three out of the five Egyptian vulture (*Neophron percnopterus*) pairs in the country along with an significant number of Griffon vultures (*Gyps fulvus*) [36,37].

Wolf attacks on hunting dogs in the DNP and adjacent areas have been increasingly reported by hunters over the last few years, raising concern over a potential rise in illegal use of poison baits. Therefore, it is of critical importance that the conflicts are properly assessed and managed.

This is the first study in Greece dealing with wolf–hunting dog interactions and one of the few in Europe. The aims of the study are (a) the preliminary assessment of wolf depredation on hunting dogs and its magnitude in a biodiversity hot spot area; (b) the investigation of conflict trends and related causes; (c) the identification of factors affecting spatial distribution of the attacks, their severity level per hunter, geographical areas, or wolf territories; and (d) the suggestion of proposals for monitoring and mitigating the conflict.

## 2. Materials and Methods

### 2.1. Study Area

**Figure 1:**
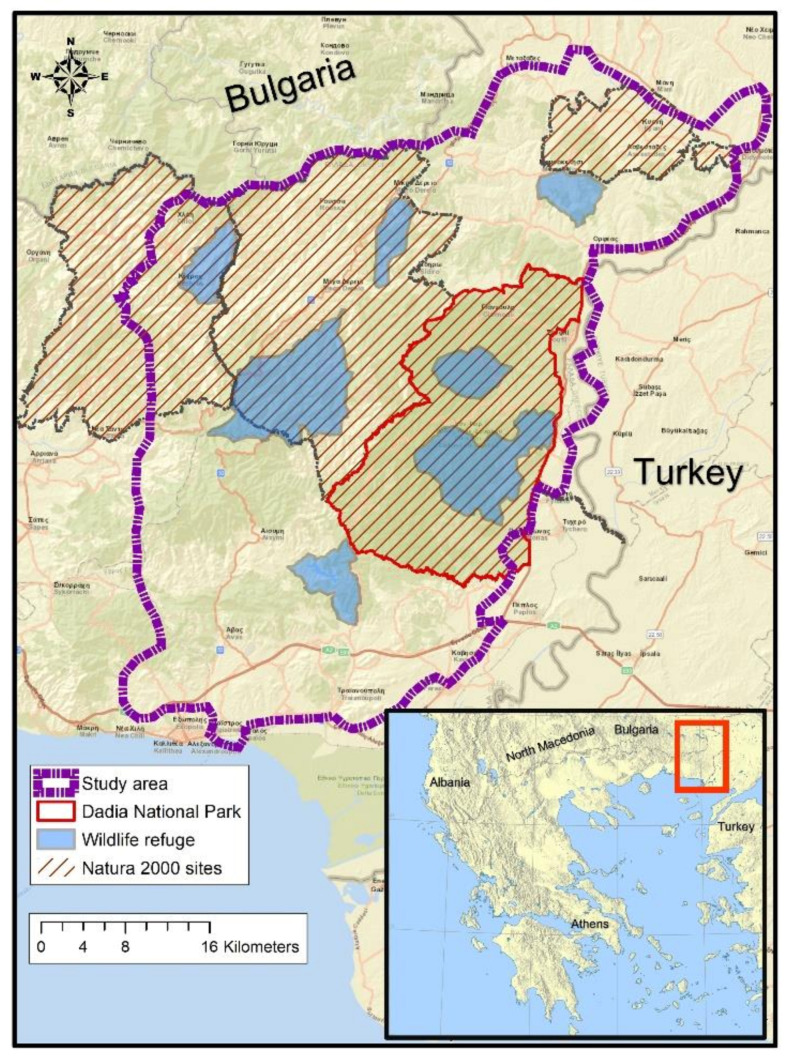
Study area.

The study area is located in north-eastern Greece on the borders with Bulgaria and Turkey. It covers approximately 2000 km^2^, including four Natura 2000 sites, among those the DNP, seven wildlife refuges, and their adjacent areas (Figure 1).

Most of the area is hilly (mean altitude = 285 m.a.s.l, range: 0–1270 m), with gentle to moderate slopes, transected by valleys with temporary streams with high landscape heterogeneity. The climate varies from Mediterranean along the coastline in the south, meso-Mediterranean in the central part, and continental in the mountainous northern part. The vegetation consists mainly of deciduous thermophilus sub-continental oak forests at lower altitudes and beech forests at higher altitudes, with scattered pine reforestations. Pure, or mixed with oak, pine forests prevail within the area of the DNP. Agroforest landscape and scrubland occupy a large part of the study area, mainly in the south-east and the north-west of the study area.

The main human activities include livestock grazing and cultivation of annual crops for grain and tobacco production [38]. Approximately 76,000 sheep, 53,500 goats, and 14,000 cattle graze inside the study area [39], with densities per village (municipal district) averaging 24.45 sheep/km^2^ (range: 0–82), 18.5 goats/km^2^ (range: 0–78), and 4 cattle/km^2^ (range: 0–45). Two wild ungulate species are present, wild boar (*Sus scrofa*) and roe deer (*Capreolus capreolus*), the latter at a minimum density of 3.6 roe deer/km^2^ [40]. Only the wild boar is hunted. The wolf population consisted of nine social units in 2019 (i.e., any territorial wolf assemblage ≥2 animals, [41]), with a summer density of 3.2–3.7 wolves/100 km^2^ and their respective territories averaging 200 km^2^ [42]. The DNP (423 km^2^) hosts one of the richest birds of prey communities in Europe [37] and a remarkable diversity of 373–413 plant, 125 butterfly, 12 amphibian, 30 reptile, and 65 mammal species [43,44], and has been a successful case study in wildlife conservation in Greece and Europe.

### 2.2. Data Collection

#### 2.2.1. Questionnaire Surveys to Hunters

No official reports on confirmed wildlife–dog interactions exist for the study area so far. Fatalities of hunting dogs are not covered by the national compensation system, except for a single private insurance initiative launched very recently from local hunting clubs (Didymoteicho hunting club; D. Vasilakis, Didymoteicho forestry service, pers. communication). Therefore, only scarce data on confirmed attacks and only from one year were available. To deal with data deficiency, we interviewed affected hunters, collecting information on dog depredation for a wide period and geographical area. Questionnaire surveys have been extensively used to collect data on large carnivore attacks on livestock [45,46,47,48,49] or hunting dogs [3,18,50].

We used non-probability convenience sampling, implementing the snowball technique, which is a respondent-driven sampling technique that allows researchers to make asymptotically unbiased estimates for socially hidden populations [51]. We asked each hunter interviewed to name others also experiencing hunting dog losses [18]. We arranged face-to-face interviews (February–July 2020) by the same person (Y.I.) to reduce systematic researcher’s bias. The questionnaire consisted of 11 sections with 68 questions related to hunting practices, hunting, and dog training areas, and 33 questions dedicated to circumstances under which each claimed wolf–dog interaction took place (Table S1). Apart from wolf–dog interactions, hunters also reported attacks to their hunting dogs from wild boar or livestock guarding dogs (LGD’s) when those were witnessed (Section S8, Table S1). We also recorded hunter opinions and beliefs on preventive methods (protective dog vests, predation risk maps) and trends on hunting practices in the study area using open questions (Section S10, Table S1). Hunting and dog training areas (i.e., polygons), as well as interaction or depredation localities (i.e., points), were mapped over Google Earth maps during the interviews.

We divided wolf–hunting dog interactions according to their outcome as follows: (a) wolf approaches or attacks without injuries; (b) injuries or fatalities. All dog interactions were evaluated on a case-by-case basis, considering all provided information including photographic material, and then classified as follows:

(a) *Verified*: (1) wolves were directly observed approaching, attacking, injuring, or killing a dog(s) and/or (2) observed close to the attack site and then characteristic wounds were evident on injured or killed dogs, like throat and neck bites or other paired incised or punctured wounds [52].

(b) *Probable*: (1) Injured or killed dogs had characteristic wounds and/or (2) when those were absent, following extensive feeding on the carcass, consumption rates were very fast (i.e., full consumption in less than one hour). Wolves can devour up to 1 kg of muscle mass per minute [53], and extensive and/or fast dog consumption is commonly reported in confirmed wolf–dog interactions [16,51].

(c) *Alleged-claimed*: Carcasses were retrieved after several days or not at all. Wolf involvement was only suspected, without any direct evidence.

Consumption was classified as follows: (a) absent; (b) partial, if a dog’s carcass had been partially eaten; and (c) complete, when the whole dog was eaten or only the head remained. Time of the year was classified as follows: (a) hare/wild boar hunting season (September 15 to January 15) and (b) training season (rest of the year). Time of the attack was classified as follows: “morning” (6 am–noon) and “afternoon” (all hours after noon). Hunters were classified into three groups according to game preferred: wild boar, hare, and wild boar and hare hunters.

#### 2.2.2. Wolf Attacks on Livestock in the Study Area

The number of compensated wolf attacks on livestock per village (municipal district) was obtained from the Hellenic Farmers Insurance Organization for the years 2010–2019 [54].

#### 2.2.3. Trophic Analysis of Wolf Scats

We collected canid scats from June 2019 to March 2020. After excluding those that might have belonged to dogs (i.e., in areas where dogs observed, dog tracks were found or livestock presence was frequent), we selected *n* = 103 wolf scats, both from summer (June–September, *n* = 63) and autumn/winter (October–February, *n* = 40). We prepared scats for analysis following the protocol and procedures described in similar analyses [55,56] and calculated% frequency of occurrence (FO%) in the sample for the following: (a) livestock (sheep, goat, cattle); (b) wild boar; (c) roe deer; (d) domestic dog; and (e) other food items (smaller mammals, reptiles, and so on).

### 2.3. Statistical Analyses

#### 2.3.1. Data Sets Used for Analyses

We combined “Verified”, “Verified- Probable”, or “All cases” (pooled events) with “All interactions” or “Injured-Killed” categories. Dog depredation levels were expressed either as event counts or as average annual loss rates (cases or dogs). Data sets and dependent variables used were specified for each analysis. The number of events and average annual loss rates were also calculated for incidents of wild boar and LGD attacks on hunting dogs, as they were repeatedly reported during the interviews.

#### 2.3.2. Distribution of Attacks in Relation to Wolf Annual Cycle

We calculated the number of days spent for hunting and dog training per month and then defined the expected number of wolf–hunting dog interactions in analogy to the duration of each period (“wolf season”) of the wolf annual cycle [57], i.e., (1) nomadic (November–April); (2) denning-pup weaning (May–July); and (3) post-weaning season (August–October). We used the Bonferroni confidence interval method [58] to calculate deviation percentages of the observed number of wolf–dog interactions from expected ones among wolf seasons [59]. When the expected proportion of usage (EUP) did not overlap with the estimated Bonferroni intervals for the observed proportion of usage (OUP), the deviations from expected interactions were considered significant at *p* < 0.01.

#### 2.3.3. Hunting Dog Breed and Age Classes’ Selection

Numeric availability for each hunting dog breed and age class was derived from our hunter sample. We grouped dog breeds into four classes: (a) “*Hellenic hound*” (*n* = 127), (b) “*Griffon hound*” (*n* = 40), (c) “*Segugio Italian hound*” (*n* = 29), and (d) “*all other hounds*” (*n* = 46) (i.e., *Beagle, Barak, Ariege, Istrian, Posavatz, Swiss, Artois, Pointer, Serbian Tricolor, Kurzhaar, Jura, Plott, Bleu de Gascogne, Dogo Argentino,* and *Porcelain*); and the age of dogs into three classes: “0–2”, “3–5”, and “≥6” years. We used the Chi-square goodness of fit test to examine if “Injured-killed” dogs for each verification class were distributed evenly among breeds or age classes. All expected subgroups were ≥5 in sample size to meet assumptions of the Chi-square test. We then used *Jacobs selectivity index* D [60] to quantify prevalence or avoidance of injury or predation for each specific breed and age class. The index ranges from −1 (strongest avoidance) to +1 (strongest preference). Indices near or equal to 0 indicate predation according to numeric availability.

#### 2.3.4. Dog Depredation Trends

We calculated trends for the period 2010–2020 for the following: (a) each verification and interaction category data set and (b) compensated wolf attacks on livestock, with the MAKESENS 1.0 software [18,61] based on the nonparametric Mann–Kendall test for the trend and the nonparametric Sen’s method for the magnitude of the trend [62]. To account for effects of dog-GPS use on dog retrieval probability, we calculated the sum of all hunters using GPS collars for any particular year (“GPSYEAR”). We then weighted the number of dog interactions per year with “GPSYEAR”. We calculated trend statistics on standardized Z-scores of both weighted and non-weighted interactions to allow for comparisons.

#### 2.3.5. Dog Depredation Risk Map—Maxent Analysis

We used the “verified-probable” cases (*n* = 76) and MAXENT species distribution modelling [63] to create a predictive risk map and define environmental co-variates that affect the spatial distribution of the attacks on hunting dogs in the study area. MAXENT uses only presence data, and the algorithm compares the attack locations [64] to all the available environmental variables. We standardized the analysis using a Bias file (i.e., a sampling effort raster map) based on the number of hunting and training effort days distributed per year (Figure S1). All Maxent parametrization, bias file creation, and model selection procedures are described in Appendix A. For each grid cell (100 × 100 m), we assigned the values of 33 environmental variables, tested for collinearity (Table S2). Categorical forest maps were derived from the “Copernicus Land Monitoring Service” [65]. We used Fragstats 4 [66] and the moving window option to estimate edge density (ED), core area/total area (CA/TA), and the Shannon diversity index (SHDI) at various radiuses. The ED takes higher values when forest patches and openings are highly mixed, and the CA/TA gives a measure of how unfragmented a forest patch remains in the predefined radiuses (Table S2).

#### 2.3.6. Association of Illegal Poison Bait Use in DNP with Dog Depredation Risk Map

We examined if 62 confirmed cases of illegal poison bait use from the WWF Greece archives (2012–2020) were spatially associated with the dog depredation risk map. Most of the cases (*n* = 55) involved poisoned working dogs, poisoned wildlife, or poison baits detected by an anti-poison dog unit or after retrieving poisoned GPS-equipped black vultures or other birds of prey (*n* = 7), [67,68,69,70,71,72]. For each case, we assigned the average dog predation risk within a radius of 1 km, classified into 10 equaled-sized classes (Bins). We then calculated the percentage distribution of poison use localities for each class.

### 2.4. Factors Related to Dog Depredation Levels

#### 2.4.1. Severity of Attacks on Hunting Dogs

We tested the effects of the following factors and covariates on the outcome of a wolf attack on hunting dogs using the “verified” × “all interactions” data set: “*Wolf season*”, “*Hunting activity* (*hunting or training*)”, “*Game hunted*”, “*Gunshot hunter reaction* (*Yes or No*)”, “*Hunter reaction other than gunshot* (*Yes or No*)”, “*Distance of dog from hunter*”, “*Number of other humans present*”, “*Number of wolves involved*”, and “*Use of GPS dog units (Yes or NO)*”. We used generalized linear models (GLMs) with an ordinal probit link function after we coded the attack outcome as an ordinal numeric multinomial dependent variable with the following states and corresponding values: (a) wolf approach without an injury = −1, (b) injury = 0, (c) killing of one dog = 1, and (d) multiple dog killings = 2.

#### 2.4.2. Levels of Hunting Dog Losses per Hunter

We used GLM analysis with a linear link function on a scaled continuous dependent variable to examine the possible effect of the following factors and covariates to the average annual rates of “injured-killed” dogs per hunter using the “verified-probable” data set: “*Type of game*”, “*Number of hunting dogs*”, “*Hunting effort* (*days*)”, “*Training effort* (*days*)”, “*Total effort*”, “*Type of dog husbandry* (*tighten, enclosed in shelter, free*)”, “*Hunter experience in years*”, and “*Participation in a hunting team*, (*Yes or no*)”. To avoid sampling bias among hunters due to variations in retrieving killed dogs, we analyzed data only from hunters that use GPS in all their dogs and only for years following purchase of the units.

#### 2.4.3. Levels of Hunting Dog Interactions per Village (Municipal District)

We spatially assigned “verified-probable” cases (*n* = 76) of all interaction types and calculated the average and cumulative predation risk with zonal statistics (ArcGIS 10.3) per village sampled during the study (*n* = 28). We then performed bivariate partial correlation controlled for village area size to examine the level and direction of correlations between the number of hunting dog interactions with “*Livestock abundance*”, “*Maxent predation risk (average and cumulative)*”, “*Hunting effort*”, and “*Number of wolf attacks on livestock*” using bootstrapping as the validation method at 2000 iterations.

#### 2.4.4. Severity of Losses per Wolf Social Unit for the Year 2019–2020

We used the “verified” and “verified-probable” number of wolf–dog interactions for the period 2019–2020 for each wolf territory (*n* = 9) identified in the same year [42]. We spatially assigned interactions based on their nearest distance from the center of summer wolf homesites. We performed GLM analysis with a negative binomial link function for count data to overcome evident overdispersion of the data sets and tested the possible effect of the following factors and covariates: “*Wolf reproduction*”, “*Number of adult wolves*”, “*Number of pups*”, “*Total wolf pack size*”, *Relative Abundance Indexes (RAI) for roe deer and wild boar*, “*Mean density of livestock species*”, “*Average predation risk*”, and “*Cumulative predation risk*”. Ungulate RAIs and wolf status per pack were derived from the 2019–2020 wolf census [42].

We did not examine in the same GLM analysis covariates correlated with coefficients > |0.5| and considered VIF collinearity criterion to be <2.5 for any variable in a model [73] and over-dispersion as non-prominent when deviance and Pearson dispersion ratios were ~1 [74]. We ranked candidate models using Akaike information criterion corrected (AICc) for small sample sizes [75]. When ΔAICc between candidate models was >2, we considered as the best model the one with the lowest AICc value [75]. Fisher’s exact test was used to examine if willingness of hunters to use protective dog vests differed depending on the type of game hunted. The Kruskal Wallis and Mann–Whitney U non-parametric tests were used to compare median values of retrieval time among the three consumption classes. All analyses were conducted using SPSS26 (IBM 2018).

## 3. Results

### 3.1. Hunting Dog Depredation Interactions per Affected Hunter

We interviewed 56 hunters (mean age = 53.2 years; range: 23–78; average hunting experience = 32 years; range: 9–53). As we focused interviewing mostly on affected hunters, the greatest majority (*n* = 51) claimed for a total of 110 wolf–hunting dog interactions involving 131 dogs for the period 2005–2020. Of those, 38% were classified as “verified”, 34% as “probable”, and 28% as “alleged-claimed” (Table 1).

**Figure 2:**
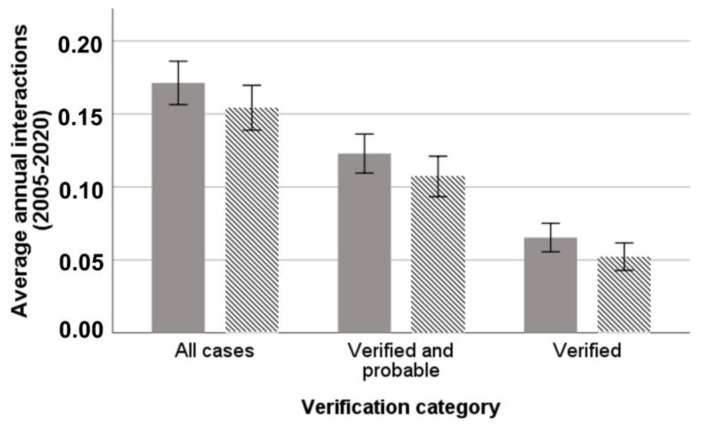
Average annual rate of dog interactions per hunter in the study area for the period 2005–2020 for each verification class. Error bars represent ±1 confidence intervals. Solid bars represent number of dogs and dashed bars represent number of events.

Of all “verified-probable” dog interactions (*n* = 94), 84% occurred in the morning (*n* = 75), 60% (*n* = 48) in the first hour after releasing, 78% (*n* = 74) were related to hunting and 22% (*n* = 20) to training of dogs, 68% (*n* = 63) to hare hunting, 28% to wild boar hunting (*n* = 26), and 4% (*n* = 3) to livestock grazing. Seventy-nine percent (79.8%) were lethal (*n* = 75), of which *n* = 62 dogs were retrieved. Of those, 8% (*n* = 5) were not consumed, 50% (*n* = 31) were partially consumed, and 42% (*n* = 26) were completely consumed. Median retrieval time (in hours) was significantly higher (Kruskal Wallis H = 16.366, d.f. = 2, *p* < 0.0001) for dogs found completely consumed (median = 1.75) compared with those found partially (median = 0.5) or not consumed (median = 0.25). Dogs killed or injured per affected hunter averaged 2.45 dogs when considering “all cases” (range 1–8 dogs, SD = 1.56, *n* = 48), 2.1 dogs for “verified- probable” (range 1–7 dogs, SD = 1.33, *n* = 39), and 1.65 dogs (range 1–4 dogs, SD = 1.86, *n* = 25) for “verified” cases. The average annual rate of dog interactions of all types experienced per hunter (*n* = 51) ranged from 0.052 to 0.171 dogs per year, depending on the verification class examined (Figure 2).

### 3.2. Monthly and Seasonal Variation of Wolf–Dog Interactions

Wolf–dog interactions peaked in late autumn and winter months, with the highest number of interactions recorded between October and December (Figure S2). Interactions were distributed disproportionally to hunting and training effort, with 39% to 46% more interactions than expected during the wolf post-weaning season (Figure S3).

### 3.3. Hunting Dog Breeds and Age Selection

Kills and injuries were not significantly distributed evenly among dog breeds when considering “all cases” (*X*^2^ = 7.915, df = 3, *p* = 0.047) or among age classes when considering “verified” cases (*X*^2^ = 10.956, df = 2, *p* = 0.004). Jacob’s selectivity index revealed a mild selection (D = 0.173) for the small- to medium-sized *Hellenic (Greek) hound*, a prominent avoidance (D = −0.411) of the larger *Griffon breed*, and a weak avoidance (D = −0.092) for the medium- to large-sized *Segugio Italiano* breed. The fourth group, which included several other less used breeds, were killed or injured according to their numeric availability (Figure S4a). Young dogs (0–2 years) were highly selected, while medium aged (3–5) or older dogs (≥6 years) were killed or injured less (D = 0.557, −0.292, and −0.3, respectively, for the “verified” data set), a pattern evident for all data sets (Figure S4b).

### 3.4. Trends of Wolf–Hunting Dog Interactions and Wolf Depredations on Livestock

**Figure 3:**
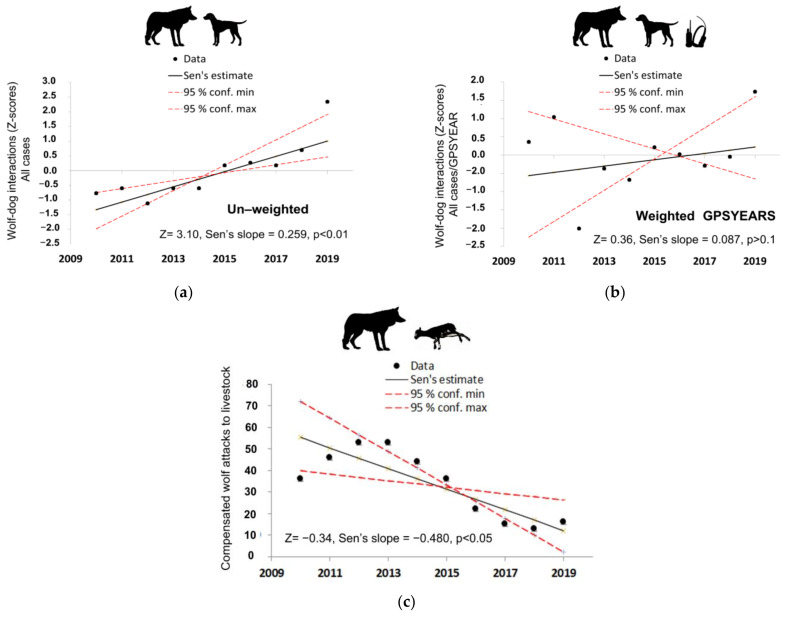
Trends for the unweighted (**a**) and weighted (**b**) with dog-GPS use (GPSYEARS) wolf–hunting dog interactions for the “all cases” verification class, and (**c**) trends of confirmed wolf attacks on livestock in the study area.

Unweighted wolf–hunting dog interaction trends for 2010–2020 were positive and significant (*p* < 0.01) for all verification classes, i.e., “all cases” (Figure 3a), “verified- probable”, and “verified” cases (Sen’s slope = 0.259, 0.279, and 0.328, respectively). Trends for weighted interactions with “GPSYEARS” were also positive, but non-significant (*p* > 0.1 for all classes) and appeared weak for the “all cases” data set (Sen’s slope = 0.087, Figure 3b) and less prominent when compared with the unweighted data for the “verified-probable” (Sen’s slope = 0.204) and “verified” datasets (Sen’s slope = 0.183). On the contrary, compensated wolf attacks to livestock followed a strong negative and significant trend during the same 10-year period (Figure 3c).

### 3.5. Wild Boar and LGDs–Hunting Dog Interactions

Wild boars were reported to have caused injuries or killing of 158 hunting dogs during the reference period, almost exclusively related to wild boar hunting (*n* = 151) and rarely to hare hunting (*n* = 7). Of those dogs attacked, 86% (*n* = 136) were injured and a lesser percentage were killed (14%, *n* = 22). Wild boar killed or injured dogs overall seven times more often compared with wolves (0.88 versus 0.13 dogs annually, respectively) for the wild boar hunter group, and two times more often for the hare/wild boar hunting group. However, these differences were mostly associated with injuries, as wild boar and wolf dog kill rates were similar for the wild boar hunter group (0.11 versus 0.1 dogs killed annually, respectively) (Figure S5). Of those hunters that responded to the relevant questions (*n* = 31), 45% also experienced losses from LGDs, averaging 2.01 dogs per hunter (range = 1–5, SD = 2.05, *n* = 14) for the whole reference period. Hunters did not report consumption of those killed dogs by livestock guarding dogs.

### 3.6. Wolf Diet Analysis

Roe deer was the most common prey found overall in scats (FO% = 47.6%), followed by wild boar (FO% = 25.2%), livestock (FO% = 22.3%), and dogs (FO% = 1.9%). Predation on roe deer involved mainly fawns (71% of all roe deer prey items). Wolves switched from young roe deer in the summer (FO% = 62.5%) to wild boar (FO% = 43.6%) during winter months, while FO% of dogs increased to 5.1% (Figure S6).

### 3.7. Maxent Dog Predation Risk Map Model Selection and Variables Involved

**Figure 4:**
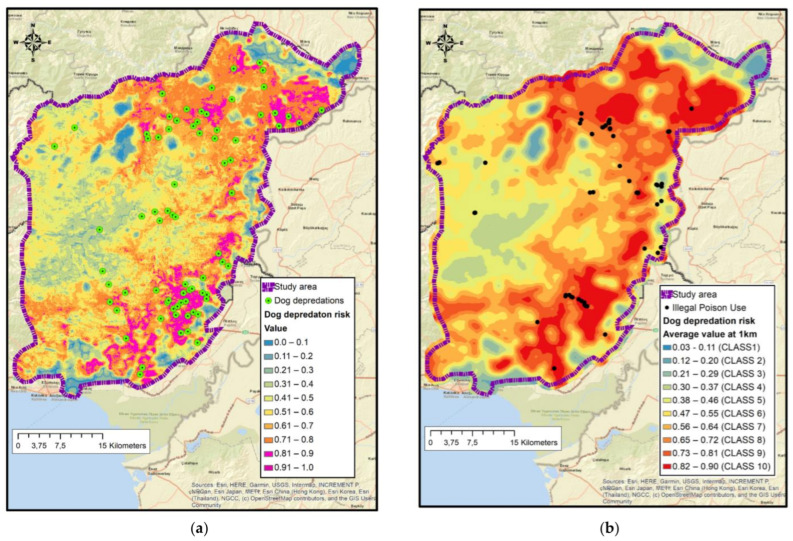
(**a**) Dog depredation events and risk map in the Dadia-Lefkimi-Soufli Forest National Park and adjacent areas. (**b**) Distribution of poisoning incidents (2012–2020) overlayed over the dog depredation risk predictive map averaged within a radius of 1 km (right). Classifications are in 10 equally sized classes.

We selected the most parsimony models (*n* = 25) with an ΔAICc < 2. After bootstrapping (*n* = 76 replicates), models attained average AUC*train* values from 0.8 to 0.838 (Table S3). The top model that scored the highest in all four transferability criteria achieved an AUC*train* of 0.825, used the FQH feature combination, and a regularization multiplier of 1.25. Models in the range of 0.80–0.90 are considered good [76]. It encompassed 10 predictor variables (Figure S7): *Altitude, Tree cover density at 500 m, Shannon diversity index at 500 m, Cattle density, Density of livestock farms, Distance from livestock farms, Forest Class, Solar radiation*, and *Forest core area index* (both for 500 and 800 m radiuses). The riskiest areas (Figure 4a) were related to the medium range of tree cover density values (15–50%), broadleaved forests, lowland areas in the 50–200 m altitudinal range, with high landscape diversity, in larger and less fragmented forest patches, at a lower to medium range of solar radiation, in areas with lower density of livestock farms, in the range 1–3 km from livestock farms, and with a low density of cattle (Figure S8, Table S4).

**Figure 5:**
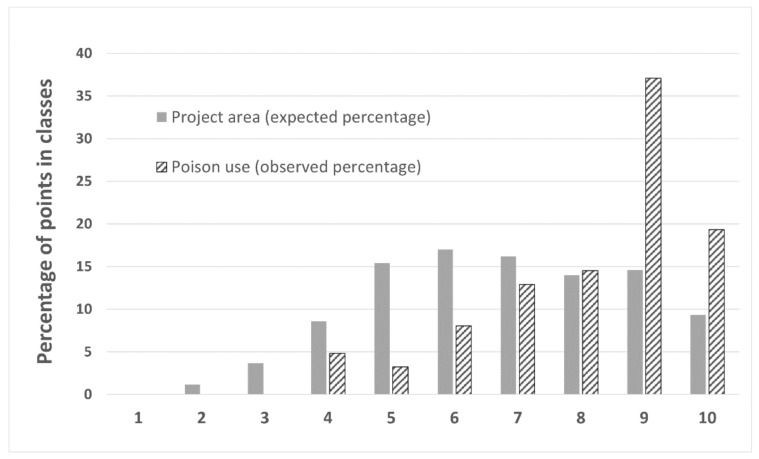
Frequency distribution of the illegal poison bait use locations over the dog predation risk classes (1–10 equal class interval).

Seventy-one percent (71%) of all poisoning incidents overlapped to the upper three equal-sized classes of the depredation risk map, covering 38% of the study area (Figure 4b and Figure 5).

### 3.8. Factors Affecting Dog Depredation Levels

#### 3.8.1. Attack Level

The best-fitted GLM model included five factors. Reaction of hunters with gunshots (shooting in the air) or with other means (shouting, calling back the dogs) and using GPS units on dogs significantly reduced the severity of an attack. An increased distance of a hunting dog from a hunter when combined with hare hunting and a higher number of wolves participating in an attack positively and significantly increased severity (Table 2).

#### 3.8.2. Hunter Level

The best model included four variables (Table 3). When hunting hare, there is a 25.4% predicted increase on the number of dogs killed or injured annually. For each hunting or training day added, there is a 0.7% decrease, or a 10% decrease in annual losses when spending 2 more weeks per year in the hunting grounds with dogs. An approximately 10% decrease is predicted per decade of hunter experience and a further decrease of 15.8% is expected when participating in a hunting team (Table 3).

#### 3.8.3. Village Level

The number of “verified-probable” interactions was strongly and positively correlated to the cumulative maxent predation risk assigned for each village (*n* = 28), negatively and moderately correlated to the number of compensated livestock attacks, and strongly and positively correlated to cumulative hunting effort. Partial correlations with livestock density were all negative, but weak and non-significant (Table 4).

#### 3.8.4. Wolf Social Unit Level

Nine wolf social units (wolf packs) were identified in the study area during the wolf monitoring in 2019 [42]: five wolf packs with verified reproduction, two wolf packs with probable reproduction, one non-reproductive wolf group, and one wolf pair. The number of “verified-probable” interactions for the year 2019–2020 varied among wolf social units (range: 0–8, *n* = 22). Three wolf units were not associated with dog interactions, three more were involved in few (*n* = 4), while the remaining three interacted with hunting dogs frequently (*n* = 18 cases) (Figure S9). The best GLM model on “verified-probable” predicts a twofold increase in the number dog interactions when reproduction is verified. The best GLM model on “Verified” interactions predicts a 2.3-fold increase in wolf–hunting dog interactions for each adult wolf added in a wolf pack (Table 5).

### 3.9. Hunter Opinions on Protective Measures and Other Information

#### 3.9.1. Use of Protective Vests

One of the methods currently available to protect hunting dogs is the use of specially designed protective vests, made of bite/puncture resistant synthetic/metal materials that minimize injury. Most hunters were positive (69.2%, *n* = 35) or almost positive (7.7%, *n* = 4), while a significant percentage (23.1%, *n* = 12) were negative on protective dog vest use. The rate of negative to positive responses was significantly higher for the hare hunters compared with wild boar hunters (Fisher’s exact test, *p* = 0.37). Hunters that were positive lost more dogs annually compared with those with negative opinion (X1 = 0.26, SD = 0.32, X2 = 0.14, SD = 0.22 respectively), but the median difference was not significant (Mann Whitney U = 81.5, *p* = 0.193). Τhe most discussed issues and doubts on dog vest use were as follows: (a) cost of the vests; (b) vest tolerance by hunting dogs during hot weather, and (c) effectiveness of vests to withstand a predatory wolf attack.

#### 3.9.2. Use of Risk Maps for Minimizing Wolf–Dog Encounter Rates

Hunters were in general reluctant and skeptical about the usefulness of predation risk maps. Forty-one percent (41%) of the respondents (18 out of 44) found some merit on risk maps, while 59% found little or no use. The most commonly expressed doubts were related to the following: (a) the untrustworthiness of risk maps as a consequence of the unpredictable nature of wolf behavior (*n* = 17); (b) their redundancy given the fact that areas with wolf attacks quickly become known (*n* = 13); (c) the lack of practicability (*n* = 6) as hunters cannot easily alternate hunting areas; (d) trust issues (*n* = 3) on those who would create risk maps; and (e) concerns of increasing illegal poison bait use.

#### 3.9.3. Hunter Practices

Hunters reported a considerable increase in wild boar hunting (61%, *n* = 28) and a consequent decrease in hare hunting (42%, *n* = 24) in the study area in the last 10 years. The reasons reported for those trends included the following: (a) increased wild boar population size, (b) use of GPS units for dogs that facilitate drive hunts, (c) sharing of costs in large hunting groups, and (d) participation of new members without prior hunting experience. Moreover, hunters reported a strong positive trend in hunting dog numbers per hunter in the study area.

## 4. Discussion

### 4.1. Dog Depredation Levels and Trends

Verified wolf attacks accounted for 38% (*n* = 50 dogs) of all claimed dog interactions reported (*n* = 131 dogs). However, a large percentage of claims (28% of all dogs) lacked any direct evidence for wolf involvement, as carcasses were not retrieved, while for the remaining cases (34%), wolf involvement was probable. Even if considering all self-perceived attacks, average dog losses per affected hunter were relatively rare. The annual rate was low even for the less conservative “verified-probable” class and even lower for the “verified” class (0.14 and 0.11 dogs respectively), being approximately one dog per decade per hunter on average. Hunting dog depredation events showed an increasing trend the last 10 years like in most areas where wolf–dog interactions were studied [8,17,18,19]. However, those were smoother or non-significant when the effect of using GPS units in detecting killed dogs was taken in account. As wolf-related conflicts are given extensive media coverage and may have a considerable negative effect on public perception of wolves [77], trends should be accurately assessed and reported correctly to the public.

### 4.2. Predatory Character of Dog Fatalities—Percentage in Wolf Diet

Most wolf–dog interactions resulted in fatalities (79%) a pattern consistent with results from Sweden (71%) [17] and Croatia (86%) [18], and had a predatory character as post-mortem consumption reached 92% of retrieved dog carcasses and in line with percentages observed in Minessota, United States (74%) [10]; Finland [16]; Sweden (72%) [17]; and Croatia (96%) [18]. However, dogs in the wolf scat sample appeared at a low frequency and in agreement with most analyses (i.e., occurrence < 5%) [1,13,78].

### 4.3. Spatial Distribution of Attacks and Related Factors

Six factors related to landscape variables and four to livestock presence and human infrastructure shaped spatial distribution of dog predation in the study area. The riskiest areas were located mostly in low altitudes (50–200 m), i.e., in more human-dominated landscapes, as commonly observed in parts of the world where wolves predate on carnivores [13]. However, those risky areas had low to medium livestock availability and density of livestock farms, as similarly observed in Wisconsin, United States [19]. In general, wolves tend to avoid proximity to areas with a high density of farms [79] especially during the daytime, a pattern also followed by hunters, in order to avoid their dogs getting killed by LGDs. Moreover, risk maximized at the range of 1–3 km from farms. Wolves may still utilize areas around livestock farms to kill stray livestock at night or to feed on livestock carcasses [56,80,81]. Keeping a relatively close, but still safe enough distance during the day may have resulted in increased encounter rates with hunting dogs at that specific range. Carcass disposal close to farms attracts wolves and is considered not only as a risk factor for wolf–livestock conflicts [81] but also as an accelerator of wolf–dog interactions [82]. In effect, wolf attacks on bear hunting dogs in Wisconsin and Michigan, United States, were exacerbated by bear baiting attracting wolves to bear hunting areas [14]. Shannon landscape diversity index was higher in risky areas and could be linked to an increased habitat suitability for many game species; that is, hares that prefer a highly edged habitat [83], wild boar for foraging and commuting [84], and roe deer that achieved highest densities in the DNP areas that combine both openings and forest [40], thus attracting both wolves and hunters. Risk was increased at the tree cover density (TCD) range between 15 and 50%, which corresponded to broadleaved forests and scrublands in the study area, favoring foraging of wild boar in winter [85]—a game and prey species for both hunters and wolves. Homogenous unfragmented forest patches consist of a critical factor for homesite selection of wolves during summer and late autumn months [86,87], where the probability of encounters with wolves increases. Moreover, those areas are used as well by wild boar as daytime refuge or foraging areas [88], and thus presumably by wild boar hunters and wolves. Dog predation risk was lower in areas with higher cattle density. Cattle provide a high biomass prey for wolves in the north-west part of the study area [54], where the least number of wolf attacks on dogs was recorded.

### 4.4. Variation among Wolf Social Units, Villages, and Factors That May Relate to Trends Observed

Not all wolf social units attacked and consumed dogs at the same intensity. Dog depredation rates were positively related to adult wolf pack size or the presence of pups. When wolf packs reproduce, their energy demand peaks owing to the high growth rate of pups [89]. In Wisconsin, United States [19], more wolf–dog interactions occurred in larger packs. Increased pack size can facilitate predation success under certain conditions [90], as GLM analysis on severity of attacks showed and as proven from a similar study in Poland [50].

However, in Finland [15], variation in wolf–dog interactions among packs was not related to pack size, but mostly to inter-pack aggression. Thus, the positive relation between adult wolf pack size and the number of attacks suggests that those are also predatory apart from agonistic—a hypothesis supported by the timing of attacks, which mostly happen during the wolf post-weaning period, in analogy to predatory attacks on livestock that peak during the same season [59,91].

Not all reproductive or large packs attacked dogs with the same intensity or—most importantly—the attacks did not occur in the past. Although no annual estimations on wolf numbers exist for the study area during the study period, apart from the year 2019 [42], as to link directly any wolf population trends with those on hunting dog attacks, wolves have always been present in the study area and reproduced at least since 1998, where a minimum of four wolf reproductive packs was recorded [92]. However, no wolf attacks on hunting dogs were reported or those were very rare in the past (i.e., before 2005). Why have attacks increased recently and not earlier in the same reproductive wolf population? Differences in depredations between areas exist despite similarities in wolf populations [14]. Several factors, other than wolf density, like low prey availability [1,3], may have predisposed packs to start predating on dogs [3,12]. Apart from prey availability, changes in vulnerability and complex interactions triggered by variations in relative abundance between different prey species may result in wolf prey switching [93].

Partial correlations at the village level revealed the possible role of both wolf–prey interactions and landscape characteristics in the trends recorded. One of the interesting results was the observed opposite trend between wolf attacks on livestock and attacks on hunting dogs in the same period. When the results of the study are combined, they indicate a possible relation of dog depredation trends with (a) declining availability and dependence of wolves on livestock and (b) seasonal variations in wolf prey selection. This initially appears to be not in line with the commonly observed pattern linking wolf predation on dogs with anthropogenic areas [13,18]. In Belarus, for example, wolf attacks on dogs and livestock followed wild boar and roe deer declines due to overhunting and poaching [3]. However, in a recent metanalysis Martins et al. [13] found a similar negative relation between consumption of carnivores in wolf diet and wolf dependence on livestock, as also observed in the study area. Thus, even if wolves may have been gradually replacing livestock with wild ungulates, as diet analysis implies, this may not be contradicted to, and be consistent with, the main assumption that attacks are caused by some shortage of prey biomass [1] at least on a seasonal basis. In Romania, similarly, severe wolf predation on dogs has also been observed after a diet shifting of wolves from livestock to wild ungulates, which currently makes up to 83% of the wolf diet [94].

Wild ungulates in the study area may have not totally substituted an extreme and fast reduction of livestock numbers of at least 60% recorded during the period 1999–2016 [95], and may partially explain the gradual decrease in the overall number of wolf attacks recorded along with the combined rise in wild ungulates densities. Nevertheless, apart from livestock and wild prey densities, husbandry and protection measures against wolf attacks play a fundamental additional role in livestock depredation levels [59,96]. However, as wolves were never extirpated in the study area, traditional husbandry methods for livestock protection (i.e., LGDs, night corals, and human supervision) were always in place and enforced from the majority of livestock producers. Most likely, the role of prey availability for both domestic and wild ungulates remains the primary factor shaping changes in wolf prey preferences along years in the study area.

Roe deer densities maybe still be not high (i.e., 3.6 ind./km^2^) when compared with other areas where it is also preyed on by wolves (i.e., 12.2–38.5 ind./km^2^ in central Italy [97,98] or Germany [99]). In Scandinavia, however, roe deer densities at three individuals/km^2^ support wolf packs [100], but in a population with a substantially lower density (i.e., <1 wolves/100 km^2^ versus 3.2–3.7 wolves/100 km^2^). Wild boar densities, though increasing, are currently unknown and have been estimated in the past to be 0.89 individuals/km^2^ [101].

Wolves shift to wild boar in winter, when most attacks on dogs occur, after the vulnerable roe deer fawn age class diminishes (i.e., 71% in summer scats) and vulnerability of young wild boar increases, a seasonal pattern also observed in other similar studies from Europe [98,102,103]. Apart from wild boar, hunting dogs may represent an alternative, locally abundant, and highly vulnerable prey during winter, also given the positive trend in their numbers in the last 10 years, as mentioned. The number of dog interactions per village was highly and positively correlated to hunting effort, further supporting that hypothesis. Additionally, wolves may have also behaviorally responded to the intensified hunting pressure of wild boar (their main prey in winter) caused by the ongoing use of GPS dog collars, and the overall increase in wild boar hunters, either by developing intra-guild interference competition with hunting dogs [20], as has also been documented between wolves and coyotes [104] or indirectly by getting attracted to wild boar drive hunts to prey on injured wild boar left by hunters [105,106]. Once wolves conceive dogs as alternative/possible prey, a strong learning process is initiated and they may start seeking them very intently whenever available [7,10] and when most vulnerable, such as, for example, during hare hunting, as shown in the analysis.

Another important change that possibly contributed to positive trends observed is the increased amount of risky habitat and overall encounter rates with hunting dogs. Predation can be affected by the landscape configuration, which influences prey vulnerability [107]. Forest cover and percentage of unfragmented forest patches, which are positively linked to predation risk, substantially increased following the abandonment of rural farming, free livestock grazing, and natural reforestation [95,108].

Apart from wolves, wild boar caused fatalities at a rate comparable to those by wolves, while injury rates were multiple times higher. Wild boar can kill and consume livestock [109,110], hunting dogs [52], or non-hunting dogs [111]. Μany unprovoked attacks on humans were associated with hunted or injured wild boar [112] and, in some cases, on accompanying dogs, perceiving them as potential predators and attacking them in defense [113], as they can alter their behavior as an effect of intensified hunting [114]. Given the opportunistic omnivorous diet of wild boar, which also scavenge οn mammal carcasses [115,116], these cases can perplex the issue of wolf–dog interactions, with responsibilities attributed falsely to wolves when lacking proper evidence on the predation event [52].

### 4.5. Implications of the Use of Risk Maps and Other Protective Measures

The spatial distribution of independent poison bait use incidents overlapped well with dog depredation risk map. However, we cannot assume an a priori causative relation between hunting dog depredations and poison incidents. Other than a direct relation, high spatial coincidence between poison bait use areas and dog predation risk maps indicates that common factors can predict both events.

The provided risk map can be either used to (a) target preventive methods for those hunters most active in the riskiest areas; (b) increase surveillance of the particular area by relevant authorities; (c) locally intensify patrols from the anti-poison dog unit; (d) warn hunters prior to hunting or training of their dogs to avoid accidental poisoning or predation, as, for example, successfully implemented in Finland using wolf telemetry locations [8]. Risk maps have also been proposed to calibrate compensation payments [19]. However, avoiding risky areas should mean a concentration of hunting in probably less favorable areas for hunting, which is expected not to be easily applicable or acceptable.

Additionally, hunters could take proactive methods at those risky areas. According to our results, dog depredation levels can be reduced when (a) hunting is performed in larger teams, (b) dogs are kept at closer distance, and (c) noise is produced with gunshots or other means. Indeed, some hunters already reported a reduction in dog fatalities by incorporating those simple methods also proposed from a recent relevant study in Poland [50]. Hare hunters appeared to be the most affected in all levels of analysis, as they hunt alone or in small teams, as also reported in Sweden [17].

Experienced hunters who spend more time in the field had less damages and probably more easily recognize risks and properly train their dogs. Interestingly, this was also true for their dogs and in line with the results from Minnesota [10]. Dog experience coming with age should be more important than physical condition as interactions between dogs and wolves are asymmetrical [1], with younger dogs behaving less cautiously in relation to wolf presence [18]. Additionally, larger breeds experienced less injures or fatalities than expected, as they probably are more capable of escaping from a wolf pursuit, or can more effectively defend themselves, as also reported from Croatia and Sweden [17,18].

While more hare hunters were affected by dog depredation (67%), wild boar hunters appear more affected when considering overall dog casualties including those by wild boar. Costs related to the purchase of dogs, veterinary care, or enforcement of protective methods (vests, collars, GPS) can be shared between team members of wild boar hunting teams. An appropriate cost analysis should be in effect to conclude.

Protective vests appear as a more cost-effective solution for wild boar hunters, as they can protect dogs from injuries and fatalities from both wild boar and wolves. However, newly designed protective vests to withstand both wild boar and wolf attacks can be bulky and heavy, as they need to incorporate both puncture resistant garments as well as metal parts for bite protection, and thus cannot be tolerated by all dogs. However, experienced wild boar hunters claimed that early training of young dogs in vest use can reduce tolerance problems.

## 5. Conclusions and Management Implications

Wolf–dog interactions at the DNP and adjacent areas were mostly of predatory character and showed a positive trend during the last 10 years. However, this trend seems partially exaggerated because of the easiness of retrieving killed dogs after the incorporation of dog-GPS collars in hunting.

The role of wolf population size and trends in the number of depredated hunting dogs needs to be evaluated in future research. Nevertheless, a positive link between wolf pack size or reproduction and the number of attacks per pack was found; however, wolf reproduction was not a prerequisite condition for depredations to occur, while not all reproductive packs killed dogs.

Positive trends were accompanied by a decreasing dependence of wolves on livestock following the collapse of traditional livestock farming. Landscape characteristics that favor predation risk possibly suggest an increased encounter rate between wolves and hunting dogs as a response to this newly formatted overlap between hunting areas and favorable wolf foraging habitat. A generalized or seasonal food shortage also cannot be excluded, but this hypothesis needs to be further justified given the rise in wild ungulate populations in the area and the high trophic dependence of wolves by roe deer and wild boar. A focus on explaining the trends should probably be given on wolf behavioral changes and encounter rates between wolves and dogs also linked to observed seasonal prey shifting.

Those shifts, from roe deer in summer to wild boar in winter, may have also predisposed wolves to be more susceptible to changes in hunting effort, hunter game preferences, and numeric availability of hunting dogs, and thus utilize more intensively that alternative prey source. Other factors related to vulnerability of dogs to wolf predation included hunter group size, hunter experience, distance of dogs from humans, and the size or age of the dogs.

Management implications should include firstly a thorough and robust verification process to correctly access all claims and exclude the cases of wild boar and LGDs attacks on hunting dogs, which were very common in the study area. All cases should be field examined from qualified veterinarians in the framework of established insurance systems and, when necessary, incorporating DNA analysis. Rigorous tests of damage verification protocols with the aid of genetics are critical in gaining public trust on compensation and insurance systems and inform for misinterpretation of cases attributed to wolves [117]. Dead livestock or other attractants to wolves and dogs should not be left in the forest to avoid increased encounter rates and wolf habituation.

Although recovery of wild boar and roe deer is ongoing in the study area, trophic stability of wolf populations and the reduction of wolf–human conflicts are achieved by increasing not only density, but also the diversity of wild ungulates [3,103]. Feasibility of reintroducing additional ungulates species previously native in the study area can be considered. Nevertheless, even in the presence of high wild ungulate density and diversity, wolves may still attack and kill dogs (predatory or agonistic attacks), and a series of proactive and passive protective measures should also be implemented, like hunting cooperatively in larger teams, intensifying control of dog movements, exploitation of risk maps for hunter warning, and extensive use of protective vests to reduce injuries or fatalities to dogs by both wolves and wild boar. The use of larger dog breeds can also be considered. Younger dogs need to be trained first in safer areas, when possible, along with older dogs to gain experience. Dogs should be trained preferably at an early age on vest use to achieve vest tolerance under diverse hunting and environmental conditions. The use of noise devices prior to hunting may also prove to be effective in risky areas. Those suggestions may be applied for reducing dog fatalities and injuries by both wolves and wild boar. Additionally, proper education of hunters about wolf biology may be beneficial for recognizing wolf signs, avoiding risky areas and understanding the rationale of preventive methods, and finally appreciating the important role of wolves in ecosystems. Overall management implications proposed may reduce conflicts and cases of illegal poison bait use that are detrimental to endangered vulture species and might affect hunters and other stakeholders (livestock breeders) whose dogs might be poisoned.

## Figures and Tables

**Table 1:** Classification of the overall number of reported dog interactions for the period 2005–2020.

Number of Dogs				
Interaction	“Verified”	“Probable”	“Alleged-Claimed”	Total
Only approach and attack	10	2	1	13
Injury	6	1	1	8
Death	34	41	35	110
Total sum	50	44	37	131

**Table 2:** Factors related to the severity of wolf attacks on hunting dogs listed in descending order of importance according to Wald Chi-square statistic (GLM with ordinal probit link function *X*^2^ likelihood ratio test = 24.945, d.f = 5, *p* < 0.0001, AICc = 75.703 for the “verified” × “all interactions” data set).

	B	Standard Error	95% Wald C.I			
			Lower	Upper	Wald *X*^2^	Sig.
Gunshots as reaction	−2.719	0.743	−4.176	−1.262	13.383	0.000
Other hunter reaction	−3.167	1.051	−5.227	−1.106	9.075	0.003
Hunting hare × Distance from hunter (km)	3.032	1.217	0.646	5.417	6.205	0.013
Number of wolves appeared	0.272	0.1293	0.019	0.525	4.430	0.035
GPS use on dogs	−0.829	0.491	−1.792	0.133	2.850	0.091

**Table 3:** Factors affecting levels of mean annual rates of hunting dog depredations per affected hunter (GLM with linear link function: *X*^2^ likelihood ratio test = 17.848, *p* = 0.001, AICc = 11.912 for the “verified-probable” data set).

Parameter	B	Std. Error	95% Wald CI				Exp(B)
			Lower	Upper	Wald *X*^2^	Sig.	
(Intercept)	0.942	0.198	0.554	1.330	22.610	0.000	2.565
Hunting and training effort (days)	−0.007	0.002	−0.011	−0.003	12.193	0.000	0.993
Hare hunting	0.226	0.093	0.043	0.409	5.879	0.015	1.254
Hunting experience (years)	−0.009	0.004	−0.016	−0.001	5.494	0.019	0.991
Participation in teams	−0.172	0.116	−0.401	0.056	2.187	0.139	0.842

**Table 4:** Partial correlations (*n* = 28), controlled for local village area size for “verified-probable” interactions with hunting dogs. Correlations significant only at Spearman r are denoted with “*” and those also significant after bootstrapping are denoted with “**”.

		Wolf Attacks to Livestock	Livestock Density	Average Dog Predation Risk	Cumulative Dog Predation Risk	Cumulative Hunting Effort
Correlation		−0.454 *	−0.235	0.374 **	0.737 **	0.704 **
Significance (two-tailed)		0.017	0.239	0.050	0.000	0.000
Bootstrap	Bias	0.061	0.022	0.015	−0.018	−0.025
	Std. Error	0.264	0.195	0.131	0.113	0.165
95% C.I	Lower	−0.745	−0.525	0.112	0.442	0.222
	Upper	0.300	0.235	0.628	0.884	0.882

**Table 5:** Factors related to the number of attacks per wolf social unit located in the study area for the year 2019–2020 for the following: (a) the “verified-probable” (GLM with negative binomial link function, likelihood ratio Chi-square = 4.693, *p* = 0.030, AICc = 38.658), and (b) the “verified” data sets (GLM with Poison log link, *X*^2^ likelihood ratio test = 17.420, *p* < 0.0001, AICc = 24.217).

Parameter	B	Std. Error	95% Wald C.I			
			Lower	Upper	Wald *X*^2^	Sig.
“Verified-Probable” interactions						
(Intercept)	−0.693	0.866	−2.391	1.004	0.641	0.423
Wolf reproduction present	2.079	1.000	0.119	4.039	4.324	0.038
“Verified” interactions						
(Intercept)	−18.767	9.1812	−36.762	−0.772	4.178	0.041
Number of adult wolves	2.334	1.046	0.283	4.385	4.975	0.026
Maxent average predation risk	27.68	14.13	−0.015	55.38	3.837	0.050

## Data Availability

Data are available from authors upon request.

## Supplementary Materials

The following are available online at https://www.mdpi.com/article/10.3390/ani11113235/s1, Table S1: Hunter questionnaire sections and information obtained; Figure S1: Sampling intensity used to create Maxent bias file; Table S2: Environmental predictors used for Maxent analysis; Figure S2: Monthly distribution of wolf–hunting dog interactions; Figure S3: Deviations from expected frequency distributions of hunting dog interactions among wolf seasons; Figure S4: Selection of dog breeds and age classes; Figure S5: Mean annual rates of dog injuries and fatalities per species involved (wolf or wild boar) for each hunter/game category; Figure S6: Seasonal frequency of occurrence (FO%) of prey items identified in a wolf scat sample from the study area; Table S3: Replicated bootstrapped models of dog predation risk in the project area; Figure S7: Jackknife tests of the training and AUC gain in the highest rank Maxent model; Figure S8: Averaged response curves for variables retained in the bootstrapped replicated best Maxent model; Table S4: Percent distribution and permutation importance of variables used in Maxent best replicated model; Figure S9: Number of “verified” and “verified and probable” dog interactions and events per wolf social unit in the project area.

## Appendix A

### Maxent Parametrization and Model Selection Procedure

The iteration threshold was increased to 5000 to allow enough time for convergence. Prevalence was kept at the default value of 0.5. Removal of duplicate presence points (i.e., in cases of multiple kills per attack in the same spot) was enabled to reduce spatial autocorrelation of data. We tested three regularization coefficient (b-multiplier) values in a reduced range and, in particular, 0.75, 1, and 1.25. B-multiplier values <1 result in a more restricted distribution maps, while those >1 tend to produce more generalized and broader maps. Giving the nature of the analysis, a predictive risk map, we considered only values closer to the default value and restricted experimentation to much smaller or larger values that could overspecialize or overgeneralize predation risk.

We used and tested only three Maxent feature classes, L (linear), Q (quadratic), and H (hinge), and their combinations (L, Q, LQ, H, and LQH) to avoid overfitting of the models and the creation of difficult to interpret response curves [118]. That particular feature combination was also the most suitable for the available number of depredation events (*n* = 76) following recommendations related to sample size, i.e., product (P) and threshold (T) features are used mainly with larger data sets [119]. We created a correlation matrix (band collection statistics, ArcGIS 10.3) to avoid the simultaneous inclusion of predictors with high bivariate correlation (Pearson r value >0.75) to reduce high collinearity in the models [120], although multicollinearity is less of a problem for MAXENT analysis when compared with other modeling considerations [64,121]. Thus, the r threshold need not to be very conservative. To select for the best Maxent model, we followed a two-stage procedure [118]. We first created preliminary non-replicated models and evaluated their importance using information criteria, and then further analyzed and classified top ranked models with cross validation (bootstrapping). To create a series of preliminary non-replicated candidate models, we used a backwards variable selection [122,123]. We run the Maxent algorithm with the whole set of variables after the removal of highly collinear variables (r > |0.75|) in the same run (baseline model).

We gradually removed variables from the baseline model according to the performance of each variable by evaluating gains in the three jackknife outputs provided (training gain, test gain, and AUC*test* gain), variable percent contribution, and permutation importance. A variable was removed when reduced model train gain, test gain, and changes in AUC values were checked. If removal resulted in a decrease in both AUC*train* and AUC*test,* we re-entered the variable in the model and continued the procedure with the next worst performing variable. In cases when AUC*train* increased while, simultaneously, AUC*test* decreased or vise-versa, and the overall change of both statistics was counterbalanced, we retained both models and continued the same procedure for each one separately. The whole procedure continued until we achieved the highest possible AUC values for each model clade. To allow for valid comparisons between preliminary models created with this procedure, we used the same background area and the same training (75% of all points) and test data points (25% of all points), by disabling the random seed option [64]. We repeated the procedure for each feature combination (L, Q, LQ, H, and LQH) and regularization parameter (0.75, 1, and 1.25).

To rank those preliminary non-replicated candidate models based on their parsimony, we used ENM Tools v.13 [124], which is a specially designed algorithm to analyze maxent outputs and select among the candidate models produced in the previous step. This function allows criterion-based model selection [125] using AIC, AICc, and BIC. We used AICc as the most suitable diagnostic criterion for our small-sized sample analysis. We considered preliminary non-replicated models with ΔAICc <2 from the best ranking model for further analysis with cross-validation with the bootstrap method, as the most appropriate method for smaller samples. The number of replications was set equal to our sample size (*n* = 76). In this way, all points are participating in bootstrapping and used in replicated models. Data partitioning during bootstrapping used 75% of all available cases for training and 25% for testing.

Model transferability and overall performance of replicated models was evaluated with four criteria, which have been used and shown to be very consistent with and related to model transferability [121,126]: AUC*test*, AUC*diff*, OR_10_, and OR*min*. AUC*diff* is the difference between AUC*train* and AUC*test*. A higher value of AUC*test* indicates a model’s increased transferability, while higher values of AUC*diff* indicate an overfitted model on training data that performs poorly when evaluated on independent testing data. OR10 is the 10% training omission rate, which is the percentage of testing points that have a suitability score lower than that of the 10% of training points with the lowest predicted suitability scores, while OR*min* is the threshold-dependent minimum training presence omission rate, defined as the percentage of testing points that have a suitability score lower than the training point with the lowest predicted suitability score [127,128]. A higher value for both metrics indicates a model’s reduced ability to identify suitable habitats in testing regions. We ranked replicated models with an ascending order of AUC*test* and descending order of AUC*diff*, OR10, and OR*min,* thus prioritizing selection for transferability, reduced overfitting, and better ability for suitable habitat recognition, respectively.

The final ranking incorporated all four criteria rankings (averaged). Given scores for each criterion ranged from 1 to *n* (i.e., *n* = the number of tested models) with lowest value correspond to best ranking model. The top model was the one that achieved the highest average ranking from all four criteria. Maxent output was at the logarithmic scale.

We controlled Maxent analysis with hunter effort by creating a special bias file based on the number of days sampled hunters spend at each part of the project area (Figure S1). Inclusion of a bias file is of paramount importance in species distribution modelling with Maxent [129]. The bias file used was in the form of raster files (ascii files) with the same resolution (cell size 100 × 100 m), extent, and geographical coordinate system as the environmental layers. Sampling effort was estimated according to how many hunting and dog training areas were mapped in the project area (sampled areas) weighted for their relative importance based on the number of days used from each hunter. The feature shapefile created in ArcGIS 10.3 was then converted to a raster file (100 × 100 m grid cell size) and merged with a similar cell-sized raster layer, which was given the value of 0 in areas where no hunting polygons were sampled (i.e., in hunting reserves). The two raster files were merged, and the final bias raster file was rescaled to achieve values ranging from 0 to 1 as the sampled intensity in the project area. Zero values were replaced with the 1^−23^ value as Maxent does not accept zero values for the bias files.

## References

[1] Butler J.R.A., Linnell J.D.C., Morrant D., Athreya V., Lescureux N., McKeown A., Gompper M.E. (2014). Dog eat dog, cat eat dog: Social-ecological dimensions of dog predation by wild carnivores. Free-Ranging Dogs and Wildlife Conservation.

[2] Boitani L., Harrington F.H., Paquet P.C. (1982). Wolf management in intensively used areas of Italy. Wolves of the World: Perspectives of Behaviour, Ecology, and Conservation.

[3] Sidorovich V.E., Tikhomirova L.L., Jędrzejewska B. (2003). Wolf Canis lupus numbers, diet and damage to livestock in relation to hunting and ungulate abundance in northeastern Belarus during 1990–2000. Wildl. Biol..

[4] Salvador A., Abad P. (1987). Food habits of a wolf population (*Canis lupus*) in León province, Spain. Mammalia.

[5] Vos J. (2000). Food habits and livestock depredation of two Iberian wolf packs (*Canis lupus signatus*) in the north of Portugal. J. Zool..

[6] Barja I. (2009). Prey and Prey-Age Preference by the Iberian Wolf *Canis lupus signatus* in a Multiple-Prey Ecosystem. Wildl. Biol..

[7] Kojola I., Ronkainen S., Hakala A., Heikkinen S., Kokko S. (2004). Interactions between wolves *Canis lupusand* dogs *C. familiarisin* Finland. Wildl. Biol..

[8] Tikkunen M., Kojola I. (2020). Does public information about wolf (*Canis lupus*) movements decrease wolf attacks on hunting dogs (*C. familiaris*)?. Nat. Conserv..

[9] Jethva B.D., Jhala Y.V. (2004). Foraging ecology, economics and conservation of Indian wolves in the Bhal region of Gujarat, Western India. Biol. Conserv..

[10] Fritts S.H., Paul W.J. (1989). Interactions of Wolves and Dogs in Minnesota. Wildl. Soc. Bull..

[11] Ruid D.B., Paul W.J., Roell B.J., Wydeven A.P., Willging R.C., Jurewicz R.L., Lonsway D.H., Wydeven A.P., Van Deelen T.R., Heske E.J. (2009). Wolf-human confl icts and management in Minnesota, Wisconsin, and Michigan. Recovery of Gray Wolves in the Great Lakes Region of the United States: An Endangered Species Success Story.

[12] Edge J.L., Beyer D.E., Belant J.L., Jordan M.J., Roell B.J. (2011). Livestock and Domestic Dog Predations by Wolves in Michigan. Hum.-Wildl. Interact..

[13] Martins I., Krofel M., Mota P.G., Álvares F. (2020). Consumption of Carnivores by Wolves: A Worldwide Analysis of Patterns and Drivers. Diversity.

[14] Bump J.K., Murawski C.M., Kartano L.M., Beyer D.E., Roell B.J. (2013). Bear-Baiting May Exacerbate Wolf-Hunting Dog Conflict. PLoS ONE.

[15] Tikkunen M., Kojola I. (2019). Hunting dogs are at biggest risk to get attacked by wolves near wolves’ territory boundaries. Mammal Res..

[16] Kojola I., Kuittinen J. (2002). Wolf Attacks on Dogs in Finland. Wildl. Soc. Bull..

[17] Backeryd J. (2007). Wolf Attacks on Dogs in Scandinavia 1995–2005: Will Wolves in Scandinavia Go Extinct If Dog Owners Are Allowed to Kill a Wolf Attacking a Dog?. Ph.D. Dissertation.

[18] Bassi E., Pervan I., Ugarković D., Kavčić K., Maksan M.T., Krofel M., Šprem N. (2021). Attacks on hunting dogs: The case of wolf–dog interactions in Croatia. Eur. J. Wildl. Res..

[19] Olson E.R., Treves A., Wydeven A.P., Ventura S.J. (2014). Landscape predictors of wolf attacks on bear-hunting dogs in Wisconsin, USA. Wildl. Res..

[20] Wierzbowska I.A., Hędrzak M., Popczyk B., Okarma H., Crooks K.R. (2016). Predation of wildlife by free-ranging domestic dogs in Polish hunting grounds and potential competition with the grey wolf. Biol. Conserv..

[21] Skordas K., Karampatzakis T., Margaritis D., Pagonis K. Preliminary data incidents reports of wolf attacks on hunting dogs in Northern Greece. Proceedings of the 2nd International Jackal Symposium Hellenic Zoological Archives.

[22] Lescureux N., Linnell J.D. (2014). Warring brothers: The complex interactions between wolves (*Canis lupus*) and dogs (*Canis familiaris*) in a conservation context. Biol. Conserv..

[23] Bisi J., Kurki S., Svensberg M., Liukkonen T. (2007). Human dimensions of wolf (*Canis lupus*) conflicts in Finland. Eur. J. Wildl. Res..

[24] Sjölander-Lindqvist A. (2009). Social-Natural Landscape Reorganised: Swedish Forest-edge Farmers and Wolf Recovery. Conserv. Soc..

[25] John F.S., Keane A., Edwards-Jones G., Jones L., Yarnell R., Jones J.P.G. (2012). Identifying indicators of illegal behaviour: Carnivore killing in human-managed landscapes. Proc. R. Soc. B Biol. Sci..

[26] Parvanov D., Stoynov E., Vangelova N., Peshev H., Grozdanov A., Delov V., Iliev Y. (2018). Vulture mortality resulting from illegal poisoning in the southern Balkan Peninsula. Environ. Sci. Pollut. Res..

[27] Sanz-Aguilar A., Sanchez-Zapata J.A., Carrete M., Benítez J.R., Ávila E., Arenas R., Donázar J.A. (2015). Action on multiple fronts, illegal poisoning and wind farm planning, is required to reverse the decline of the Egyptian vulture in southern Spain. Biol. Conserv..

[28] Skartsi T., Dobrev V., Oppel S., Kafetzis A., Kret E., Karampatsa R., Saravia V., Bounas T., Vavylis D., Sidiropoulos L. (2014). Assessment of the Illegal Use of Poison in Natura 2000 Sites for the Egyptian Vulture in Greece and Bulgaria during the Period 2003–2012. Technical Report under Action A3 of the LIFE+ Project B. The Return of the Neophron (LIFE10 NAT/BG/000152).

[29] Mateo-Tomás P., Olea P.P., Sánchez-Barbudo I.S., Mateo R. (2012). Alleviating human-wildlife conflicts: Identifying the causes and mapping the risk of illegal poisoning of wild fauna. J. Appl. Ecol..

[30] Demerdzhiev D., Hristov H., Dobrev D., Angelov I., Kurtev M. (2014). Long-term population status, breeding parameters and limiting factors of the Griffon vulture (*Gyps fulvus* Hablizl, 1783) population in the Eastern Rhodopes, Bulgaria. Acta Zool. Bulg..

[31] Velevski M., Nikolov S.C., Hallmann B., Dobrev V., Sidiropoulos L., Saravia V., Tsiakiris R., Arkumarev V., Galanaki A., Kominos T. (2015). Population decline and range contraction of the Egyptian Vulture *Neophron percnopterus* in the Balkan Peninsula. Bird Conserv. Int..

[32] Oppel S., Dobrev V., Arkumarev V., Saravia V., Bounas A., Kret E., Skartsi T., Velevski M., Stoychev S., Nikolov S.C. (2016). Assessing the effectiveness of intensive conservation actions: Does guarding and feeding increase productivity and survival of Egyptian Vultures in the Balkans?. Biol. Conserv..

[33] Sakellari M., Xirouchakis S., Baxevani K., Probonas M. (2016). Wildlife poisoning in Crete and the intentions of local interest groups to engage in anti-poisoning actions. Biodiversity.

[34] Ntemiri K., Saravia V., Angelidis C., Baxevani K., Probonas M., Kret E., Mertzanis Y., Iliopoulos Y., Georgiadis L., Skartsi D. (2018). Animal mortality and illegal poison bait use in Greece. Environ. Monit. Assess..

[35] Vasilakis D.P., Whitfield D.P., Schindler S., Poirazidis K.S., Kati V. (2016). Reconciling endangered species conservation with wind farm development: Cinereous vultures (*Aegypius monachus*) in south-eastern Europe. Biol. Conserv..

[36] Skartsi T., Vasilakis D., Elorriaga J., Catsadorakis G., Källander H. (2010). Population trends and conservation of vultures in the National Park of Dadia—Lefkimi—Soufli forest. The Dadia—Lefkimi—Soufli Forest National Park, Greece: Biodiversity, Management and Conservation.

[37] Poirazidis K., Schindler S., Kakalis E., Ruiz C., Bakaloudis D.E., Scandolara C., Eastham C., Hristov H., Catsadorakis G. (2011). Population Estimates for the Diverse Raptor Assemblage of Dadia National Park, Greece. Ardeola.

[38] Liarikos C., Catsadorakis G., Källander H. (2010). Development trajectories and prospects in the Dadia-Lefkimi-Soufli Forest National Park. The Dadia—Lefkimi—Soufli Forest National Park, Greece: Biodiversity, Management and Conservation.

[39] Hellenic National Statistical Authority (2012). Data on Livestock Availability per Local Community in the Project Area for the Year 2012.

[40] Zakkak S., Mola M., De Gooijer T., Dijk P., Halivelentzios A., Giannakidis G. Population Estimates of Roe Deer in the Dadia-Lefkimi-Soufli National Park. Proceedings of the 14th International Congress on the Zoogeography and Ecology of Greece and Adjacent Regions.

[41] Reinhardt I., Kluth G., Nowak S., Mysłajek R.W. (2015). Standards for the Monitoring of the Central European Wolf Population in Germany and Poland.

[42] Iliopoulos Y., Zakkak S., Skartsi D. (2021). Summer Wolf Population Size Estimation in the Dadia-Lefkimi-Soufli Forest National Park and Adjacent Areas by Using an Hierarchical Multimethod Approach (Habitat Modelling, Sign Surveys and Camera Trapping).

[43] Catsadorakis G., Catsadorakis G., Källander H. (2010). The Dadia-Lefkimi-Soufli National Park, Greece: Biodiversity, Management and Conservation-Introduction. The Dadia-Lefkimi-Soufli National Park, Greece: Biodiversity, Management and Conservation.

[44] Zografou K., Kati V., Grill A., Wilson R.J., Tzirkalli E., Pamperis L.N., Halley J.M. (2014). Signals of Climate Change in Butterfly Communities in a Mediterranean Protected Area. PLoS ONE.

[45] Rigg R., Finďo S., Wechselberger M., Gorman M.L., Sillero-Zubiri C., Macdonald D.W. (2011). Mitigating carnivore–livestock conflict in Europe: Lessons from Slovakia. Oryx.

[46] Dondina O., Meriggi A., Dagradi V., Perversi M., Milanesi P. (2015). Wolf predation on livestock in an area of northern Italy and prediction of damage risk. Ethol. Ecol. Evol..

[47] Khorozyan I., Soofi M., Ghoddousi A., Hamidi A.K., Waltert M. (2015). The relationship between climate, diseases of domestic animals and human-carnivore conflicts. Basic Appl. Ecol..

[48] Khorozyan I., Ghoddousi S., Soufi M., Soofi M., Waltert M. (2018). Cattle selectivity by leopards suggests ways to mitigate human–leopard conflict. Ecol. Evol..

[49] Khan T.U., Luan X., Ahmad S., Mannan A., Khan W., Khan A.A., Khan B.U., Din E.U., Bhattarai S., Shah S. (2019). Status and Magnitude of Grey Wolf Conflict with Pastoral Communities in the Foothills of the Hindu Kush Region of Pakistan. Animals.

[50] Haidt A., Gawryś R., Szewczyk M. (2021). Human Decision-Making as a Key Factor in the Risk of Wolf–Dog Interactions during Outdoor Activities. Animals.

[51] Salganik M.J., Heckathorn D.D. (2004). 5. Sampling and Estimation in Hidden Populations Using Respondent-Driven Sampling. Sociol. Methodol..

[52] Mariacher A., Fanelli R., Garofalo L., Perfetti G., Lorenzini R., Fico R. (2019). Who is the killer? Barking up the wrong tree. Mammalia.

[53] Wilmers C.C., Stahler D.R. (2002). Constraints on active-consumption rates in gray wolves, coyotes, and grizzly bears. Can. J. Zool..

[54] ELGA (2020). Confirmed Cases of Bear and Wolf Attacks on Livestock in Greece for the Years 2010–2019.

[55] Bassi E., Donaggio E., Marcon A., Scandura M., Apollonio M. (2012). Trophic niche overlap and wild ungulate consumption by red fox and wolf in a mountain area in Italy. Mamm. Biol..

[56] Petridou M., Youlatos D., Lazarou Y., Selinides K., Pylidis C., Giannakopoulos A., Kati V., Iliopoulos Y. (2019). Wolf diet and livestock selection in central Greece. Mammalia.

[57] Vila C., Urios V., Castroviejo J., Carbyn L.N., Fritts S.H., Seip D.R. (1995). Observations on the daily activity patterns in the Iberian wolf. Ecology and Conservation of Wolves in a Changing World.

[58] Meriggi A., Lovari S. (1996). A Review of Wolf Predation in Southern Europe: Does the Wolf Prefer Wild Prey to Livestock?. J. Appl. Ecol..

[59] Iliopoulos Y., Sgardelis S., Koutis V., Savaris D. (2009). Wolf depredation on livestock in central Greece. Mammal Res..

[60] Jacobs J. (1974). Quantitative measurement of food selection. Oecologia.

[61] Salmi T., Määttä A., Anttila P., Ruoho-Airola T., Amnell T. (2002). Detecting Trends of Annual Values of Atmospheric Pollutants by the Mann-Kendall Test and Sen’s Slope Estimates—The Excel Template Application MAKESENS.

[62] Gilbert R.O. (1987). Statistical Methods for Environmental Pollution Monitoring.

[63] Elith J., Phillips S.J., Hastie T., Dudík M., Chee Y.E., Yates C.J. (2011). A statistical explanation of MaxEnt for ecologists. Divers. Distrib..

[64] Rostro-García S., Tharchen L., Abade L., Astaras C., Cushman S.A., Macdonald D.W. (2016). Scale dependence of felid predation risk: Identifying predictors of livestock kills by tiger and leopard in Bhutan. Landsc. Ecol..

[65] Copernicus Land Monitoring Service. https://land.copernicus.eu/.

[66] McGarigal K., Cushman S.A., Ene E. (2012). FRAGSTATS v4: Spatial Pattern Analysis Program for Categorical and Continuous Maps.

[67] Kret E., Vavylis D., Saravia V., Ntemiri Κ. (2015). Poison Bait Detection with Specially Trained Dogs in Thrace and Central Greece Annual Report 2014 Life+ Project “The Return of the Neophron” Life10 nat/bg/000152 July 2015.

[68] Vavylis D., Kret E., Saravia V., Ntemiri Κ. (2016). Poison Bait Detection with Specially Trained Dogs in Thrace and Central Greece, Annual Report 2015.

[69] Vavylis D., Kret E., Saravia V., Ntemiri Κ. (2016). Poison Bait Detection with Specially Trained Dogs in Thrace and Central Greece, Annual Report 2016.

[70] Kret E. (2017). Poison Bait Detection with Specially Trained Dog, Annual Report 2017.

[71] Kret E., Vavylis D., Saravia V. (2019). Poison Bait Detection with Specially Trained Dogs in Thrace and Central Greece, Annual Report 2018.

[72] Kret E., Vavylis D., Saravia V. (2021). Poison Bait Detection with Specially Trained Dogs in Thrace and Central Greece and Epirus, Annual Report 2020.

[73] Johnston R., Jones K., Manley D. (2018). Confounding and collinearity in regression analysis: A cautionary tale and an alternative procedure, illustrated by studies of British voting behaviour. Qual. Quant..

[74] McCullagh P., Nelder J.A. (1989). Generalized Linear Models.

[75] Burnham K.P., Anderson D.R. (2002). Model Selection and Multimodel Inference: A Practical Information-Theoretic Approach.

[76] Elith J., Ferson S., Burgham M. (2000). Quantitative methods for modeling species habitat: Comparative performance and an application to Australian plants. Quantitative Methods for Conservation Biology.

[77] Delibes-Mateos M. (2020). Wolf Media Coverage in the Region of Castilla y León (Spain): Variations over Time and in Two Contrasting Socio-Ecological Settings. Animals.

[78] Mengüllüoğlu D., Ilaslan E., Emir H., Berger A. (2019). Diet and wild ungulate preferences of wolves in northwestern Anatolia during winter. PeerJ.

[79] Carricondo-Sanchez D., Zimmermann B., Wabakken P., Eriksen A., Milleret C., Ordiz A., Sanz-Pérez A., Wikenros C. (2020). Wolves at the door? Factors influencing the individual behavior of wolves in relation to anthropogenic features. Biol. Conserv..

[80] Migli D., Youlatos D., Iliopoulos Y. (2005). Winter food habits of wolves in central Greece. J. Biol. Res..

[81] Mohammadi A., Kaboli M., Sazatornil V., López-Bao J.V. (2019). Anthropogenic food resources sustain wolves in conflict scenarios of Western Iran. PLoS ONE.

[82] Newsome T., Fleming P., Dickman C.R., Doherty T.S., Ripple W.J., Ritchie E.G., Wirsing A.J. (2017). Making a New Dog?. Bioscience.

[83] Cardarelli E., Meriggi A., Brangi A., Vidus-Rosin A. (2011). Effects of arboriculture stands on European hare Lepus europaeus spring habitat use in an agricultural area of northern Italy. Acta Thériol..

[84] Thurfjell H., Ball J.P., Åhlén P.-A., Kornacher P., Dettki H., Sjöberg K. (2009). Habitat use and spatial patterns of wild boar *Sus scrofa* (L.): Agricultural fields and edges. Eur. J. Wildl. Res..

[85] Fonseca C. (2008). Winter habitat selection by wild boar Sus scrofa in southeastern Poland. Eur. J. Wildl. Res..

[86] Iliopoulos Y., Youlatos D., Sgardelis S. (2013). Wolf pack rendezvous site selection in Greece is mainly affected by anthropogenic landscape features. Eur. J. Wildl. Res..

[87] Sazatornil V., Rodríguez A., Klaczek M., Ahmadi M., Álvares F., Arthur S., Blanco J.C., Borg B.L., Cluff D., Cortés Y. (2016). The role of human-related risk in breeding site selection by wolves. Biol. Conserv..

[88] Herrero J., Rodrigues P., García-Serrano A., Prada C., Giménez-Anaya A., Ayala R., Fernández-Arberas O., Fonseca C. (2016). Habitat use by wild boar Sus scrofa in Moncayo Nature Park, Spain. Pirineos.

[89] Oftedal O.T., Gittleman J.L., Gittleman J.L. (1989). Patterns of energy output during reproduction in carnivores. Carnivore Behavior, Ecology, and Evolution.

[90] MacNulty D.R., Smith D.W., Mech L.D., Vucetich J.A., Packer C. (2012). Nonlinear effects of group size on the success of wolves hunting elk. Behav. Ecol..

[91] Treves A., Jurewicz R.R., Naughton-Treves L., Rose R.A., Willging R.C., Wydeven A.P. (2002). Wolf depredation on domestic animals in Wisconsin, 1976–2000. Wildl. Soc. Bull..

[92] Iliopoulos Y. (1999). Distribution and Population Estimates of the Wolf in Greece, Project Life “Lycos” NAT97-GR04249: Conservation of the Wolf (Canis lupus L.) and Its Habitats in Greece.

[93] Garrott R.A., Bruggeman J.E., Becker M.S., Kalinowski S.T., White P.J. (2007). Evaluating prey switching in wolf–ungulate systems. Ecol. Appl..

[94] Sin T., Gazzola A., Chiriac S., Rîșnoveanu G. (2019). Wolf diet and prey selection in the South-Eastern Carpathian Mountains, Romania. PLoS ONE.

[95] Poirazidis K., Kapsalis L., Kret E., Korakis G., Vassilakis D., Skartsi D. Diachronic Recording and Mapping of the Change of Grazing Capacity in the National Park Dadia-Lefkimmi-Soufli. Proceedings of the 9th Conference of the Hellenic Range and Pasture Society.

[96] Janeiro-Otero A., Newsome T.M., Van Eeden L.M., Ripple W.J., Dormann C.F. (2020). Grey wolf (*Canis lupus*) predation on livestock in relation to prey availability. Biol. Conserv..

[97] Mattioli L., Capitani C., Avanzinelli E., Bertelli I., Gazzola A., Apollonio M. (2004). Predation by wolves (*Canis lupus*) on roe deer (*Capreolus capreolus*) in north-eastern Apennine, Italy. J. Zool..

[98] Torretta E., Serafini M., Imbert C., Milanesi P., Meriggi A. (2017). Wolves and wild ungulates in the Ligurian Alps (Western Italy): Prey selection and spatial-temporal interactions. Mammalia.

[99] Holzapfel M., Wagner C., Kluth G., Reinhardt I., Ansorge H. (2011). Feeding ecology of the wolf Canis lupus in Germany—Results from the last ten years. Beiträge Jagd. Wildforsch..

[100] Sand H., Eklund A., Zimmermann B., Wikenros C., Wabakken P. (2016). Prey Selection of Scandinavian Wolves: Single Large or Several Small?. PLoS ONE.

[101] Tsachalidis E., Hadjisterkotis E. (2009). Current distribution and population status of wild boar (*Sus scrofa* L.) in Greece. Acta Silv. Lignaria Hung..

[102] Meriggi A., Dagradi V., Dondina O., Perversi M., Milanesi P., Lombardini M., Raviglione S., Repossi A. (2015). Short-term responses of wolf feeding habits to changes of wild and domestic ungulate abundance in Northern Italy. Ethol. Ecol. Evol..

[103] Imbert C., Caniglia R., Fabbri E., Milanesi P., Randi E., Serafini M., Torretta E., Meriggi A. (2016). Why do wolves eat livestock? Factors influencing wolf diet in northern Italy. Biol. Conserv..

[104] Ballard W.B., Carbyn L.N., Smith D.W., Mech L.D., Boitani L. (2003). Wolf Interactions with Non-Prey. Wolves: Ecology, Behavior and Conservation.

[105] Galaverni M., Palumbo D., Fabbri E., Caniglia R., Greco C., Randi E. (2012). Monitoring wolves (Canis lupus) by non-invasive genetics and camera trapping: A small-scale pilot study. Eur. J. Wildl. Res..

[106] Viola P., Adriani S., Rossi C., Franceschini C., Primi R., Apollonio M., Amici A. (2021). Anthropogenic and Environmental Factors Determining Local Favourable Conditions for Wolves during the Cold Season. Animals.

[107] Kauffman M.J., Varley N., Smith D.W., Stahler D.R., MacNulty D.R., Boyce M. (2007). Landscape heterogeneity shapes predation in a newly restored predator?prey system. Ecol. Lett..

[108] Triantakonstantis D.P., Kollias V.J., Kalivas D.P. (2006). Forest Re-growth Since 1945 in the Dadia Forest Nature Reserve in Northern Greece. New For..

[109] Pavlov P., HoneB J. (1982). The Behaviour of Feral Pigs, Sus scrofa, in Flocks of Lambing Ewes. Wildl. Res..

[110] Choquenot D., Lukins B., Curran G. (1997). Assessing Lamb Predation by Feral Pigs in Australia’s Semi-Arid Rangelands. J. Appl. Ecol..

[111] Ingendaay C., Burger M., Linzmann H., Brunnberg L. (2008). Injuries in the dog due to wild boar. Kleintierpraxis.

[112] Mayer J., Armstrong J.B., Gallagher G.R. (2013). Wild Pig Attacks on Humans. Wildlife Damage Management Conferences—Proceedings.

[113] Wilson C.J. (2008). Feral Wild Boar in England: Status, Impact and Management.

[114] Thurfjell H., Spong G., Ericsson G. (2013). Effects of hunting on wild boarSus scrofabehaviour. Wildl. Biol..

[115] Selva N., Jędrzejewska B., Jędrzejewski W., Wajrak A. (2005). Factors affecting carcass use by a guild of scavengers in European temperate woodland. Can. J. Zool..

[116] Ballari A.S., Barrios-Garcia M.N. (2013). A review of wild boar Sus scrofa diet and factors affecting food selection in native and introduced ranges. Mamm. Rev..

[117] López-Bao J.V., Frank J., Svensson L., Åkesson M., Langefors Å. (2017). Building public trust in compensation programs through accuracy assessments of damage verification protocols. Biol. Conserv..

[118] Mikkelsen L., Rigét F.F., Kyhn L.A., Sveegaard S., Dietz R., Tougaard J., Carlström J.A.K., Carlén I., Koblitz J.C., Teilmann J. (2016). Comparing Distribution of Harbour Porpoises (*Phocoena phocoena*) Derived from Satellite Telemetry and Passive Acoustic Monitoring. PLoS ONE.

[119] Phillips S.J., Anderson R.P., Dudík M., Schapire R.E., Blair M.E. (2017). Opening the black box: An open-source release of Maxent. Ecography.

[120] Dormann C.F., Elith J., Bacher S., Buchmann C., Carl G., Carré G., Marquéz J.R.G., Gruber B., Lafourcade B., Leitão P.J. (2013). Collinearity: A review of methods to deal with it and a simulation study evaluating their performance. Ecography.

[121] Merow C., Smith M.J., Silander J.A. (2013). A practical guide to MaxEnt for modeling species’ distributions: What it does, and why inputs and settings matter. Ecography.

[122] Li Y., Li M., Li C., Liu Z. (2020). Optimized Maxent Model Predictions of Climate Change Impacts on the Suitable Distribution of Cunninghamia lanceolata in China. Forests.

[123] Zeng Y., Low B.W., Yeo D.C. (2016). Novel methods to select environmental variables in MaxEnt: A case study using invasive crayfish. Ecol. Model..

[124] Warren D.L., Glor R., Turelli M. (2010). ENMTools: A toolbox for comparative studies of environmental niche models. Ecography.

[125] Warren D.L., Seifert S.N. (2011). Ecological niche modeling in Maxent: The importance of model complexity and the performance of model selection criteria. Ecol. Appl..

[126] Low B.W., Zeng Y., Tan H.H., Yeo D.C. (2021). Predictor complexity and feature selection affect Maxent model transferability: Evidence from global freshwater invasive species. Divers. Distrib..

[127] Peterson A.T., Soberón J., Pearson R.G., Anderson R.P., Martínez-Meyer E., Nakamura M., Araújo M.B. (2011). Ecological Niches and Geographic Distributions, Monographs in Population Biology 49.

[128] Radosavljevic A., Anderson R.P. (2014). Making better Maxent models of species distributions: Complexity, overfitting and evaluation. J. Biogeogr..

[129] Kramer-Schadt S., Niedballa J., Pilgrim J.D., Schröder B., Lindenborn J., Reinfelder V., Stillfried M., Heckmann I., Scharf A.K., Augeri D.M. (2013). The importance of correcting for sampling bias in MaxEnt species distribution models. Divers. Distrib..

