# Supplementary material for: Wolf–Hunting Dog Interactions in a Biodiversity Hot Spot Area in Northern Greece: Preliminary Assessment and Implications for Conservation in the Dadia-Lefkimi-Soufli Forest National Park and Adjacent Areas
Source: Animals (Basel). 2021 Nov 12;11(11):3235. doi: 10.3390/ani11113235 (PMC8614248; doi:10.3390/ani11113235)
Supplement: Supplementary file 1 [file animals-11-03235-s001.zip › animals-1428236-supplementary.pdf]

Supplementary material

Table S1. Hunter questionnaire sections and relevant information collected.

Section	Description and related information collected
1	Date, place of interview, type of correspondence
2	Name, age, profession, place of residence, contact details of correspondent
3	Game species hunted (hare, wild boar, both, other), hunters group size (number)
4	Mapping of hunting and dog training areas (as polygons over Google maps), reporting of hunting and training effort in days per month of the year
5	Number of hunting dogs used breeds and age per individual hunter. Information on dog husbandry (type of shelter used for hunting dogs (tighten, enclosed in shelter, free)
6	Use of GPS collars, number of devices used (percentage of dogs equipped), frequency of use (no use, partial, on all occasions), years since first use
7	Protective dog vests use (percentage of dogs equipped), frequency of use (no use, partial, on all occasions)
8	Aggregated cases of wolf wild boar and livestock guarding dog for each interaction category (approaches, injuries, fatalities) during the last 10 years, distributed per year.
9	Detailed description of interactions with hunting dogs for each case : Date, time of the day, type of activity (train or hunt), time interval between onset of activity and attack, dog behavior (barking or other), number of dogs present, number of humans present, distance of incident from hunter, reaction of the dog, distress noises heard (yes/no, description), reaction of hunter (gunshot to wolves or in the air, calling dogs, other/description), Dog GPS in use (yes / no), dog breed of dog(s), sex and age of dog (s), outcome of attacks (no injury, injury, death), retrieval time of carcasses (in minutes), wounds observed (type, and location in the carcass / injured dog), carcass consumption patterns (type, extent, location in body parts), presence of livestock in the area (yes / no, distance), presence of livestock guarding dogs (yes /no, distance), presence of known offal sites (permanent or temporal).
10	Attitudes and views in relation to the issue of wolf attacks on hunting dogs: beliefs on causes (open question), interest on dog protective vest and risk maps use to alleviate conflicts (positive, almost positive, negative), causes on opposition of using vests and risk maps (open question),
11	Presence, density and population trends of wild boar, roe deer and livestock (decrease, increase, stable), trends on hunting trends in the study area the last 10 years (wild boar hunting, hare hunting: decrease, increase, stable), trends on the number of hunting dogs used per hunter.

Figure S1

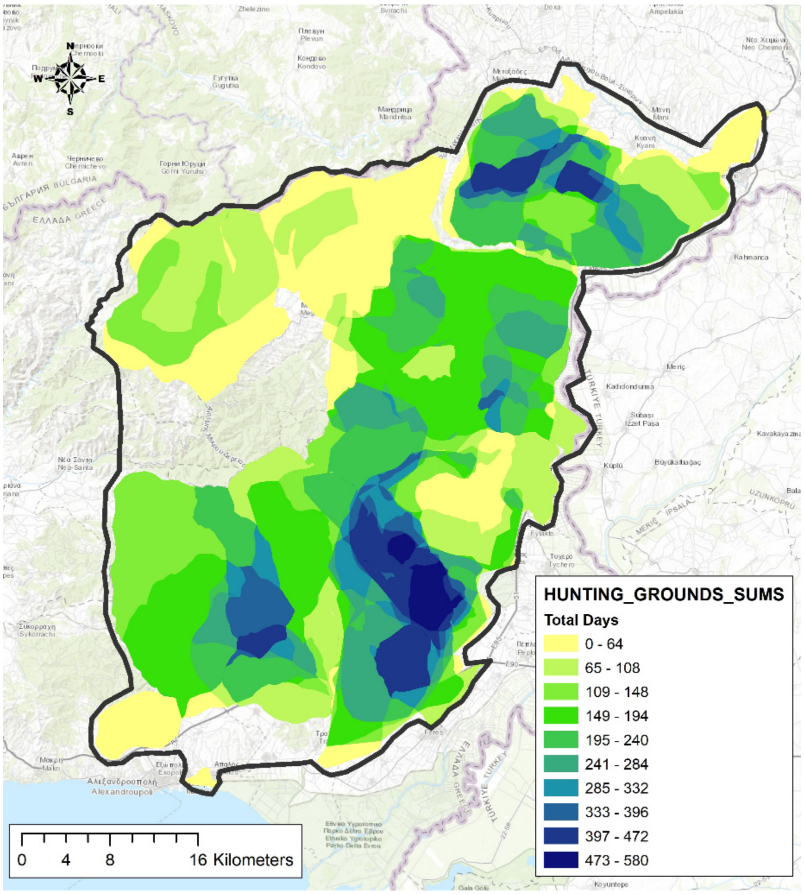

Figure S1. Sampling intensity expressed as the total days spend by sampled hunters (n=56) in the study area used to create Maxent bias file. Large unoccupied area in the center-west of the study area coincides with the large hunting reserve (prohibition of hunting) of "Kallithea".

Table S2. Environmental predictors of "verified-probable" dog-interactions used for Maxent analysis.

Code Name	Description (all raster's resampled at 100X100m grid cell size)	Source
**ALT**	Altitude (meters)	DTM, Copernicus raster map[1]
**SLOPE**	Slope (degrees)	Extracted from DTM
**SLOPE100**	Average slope at 100m radius	Extracted from DTM/Spatial analyst ArcGIS 10.3/Focal statistics
**SLOPE500**	Average slope at 500m radius	
**SLOPE1000**	Average slope at 1000m radius	
**SOLAR500AVE**	Mean yearly cumulative solar radiation at 500m radius	
**FORESTYPE**	Main landcover category: No forest (0), Broadleaved Forest (1), Coniferous Forest (2)	Copernicus thematic raster map
**CORINE**	Corine land use classes (2018)	
**TCD100**	Tree cover density- proportional tree canopy coverage at 100m radius	Extracted from Copernicus thematic map/Spatial analyst ArcGIS 10.3 -Focal statistics
**TCD500**	Tree cover density- proportional tree canopy coverage at 500m radius	
**TCD1000**	Tree cover density- proportional tree canopy coverage at 1000m radius	
**ED500**	Edge density between forest and forest openings at 500m radius	Extracted from Copernicus thematic maps with Fragstats 4 software[2] - moving window analysis
**ED1000**	Edge density between forest and no forest at 1000m radius	
**CA/TA 500**	Core Area/Total Area of forest at 500m radius	
**CA/TA 800**	Core Area/Total Area of forest at 800m radius	
**SGHI500**	Shannon diversity index at 500m radius	
**SGHI1000**	Shannon diversity index at 1000m radius	
**WATERDST**	Distance from permanent water in meters	Permanent water digitized from national military maps of 50:000 scale/ Spatial analyst ArcGIS 10.3 - Euclidean distance
**REMDST**	Distance from ravines (both permanent and temporary)	Extracted from Copernicus thematic DTM map/Spatial analyst ArcGIS 10.3 -Euclidean distance
**ROADSDST**	Distance from any road in meters	Roads (gravel, dirt roads) digitized from Google earth maps/ Spatial analyst ArcGIS 10.3 -Euclidean distance
**ROADSDNS**	Road network density (m/m^2)	Roads (gravel, dirt roads) digitized from Google earth maps/ Spatial analyst ArcGIS 10.3 with Kernel Density estimator of the overall road network (m/m^2) at a default radius
**VILLAGEDST**	Distance from villages (meters)	Digitized from Google earth maps/ Spatial analyst ArcGIS 10.3 -Euclidean distance
**PROTECTEDDST**	Distance from hunting reserves/protected areas (meters)	Thematic maps/ Spatial analyst ArcGIS 10.3 -Euclidean distance
**FARMSDST**	Distance from active livestock farms in meters	Digitized from Google earth maps and in the field/ Spatial analyst ArcGIS 10.3-Euclidean distance in meters
**FARMDNS**	Kernel Density of active Farms / km^2	Digitized from Google earth maps and in the field/ Spatial analyst ArcGIS 10.3- Kernel Density estimator ($Farms/m^2$) within an average minimum grazing distance of 2 km
**CATTLE**	Number of cattle	Data on livestock availability from the national statistical authority per municipal district (village) [3]
**CATTLEDNS**	Cattle/km^2	
**GOAT**	Number of goats	
**GOATDNS**	Goats/km^2	
**SHEEP**	Number of sheep	
**SHEEPDNS**	Sheep/km^2	
**TOTALLVST**	Number of livestock heads (all species)	
**TOTALVSTDNS**	Density of livestock heads (all species)	

Figure S2

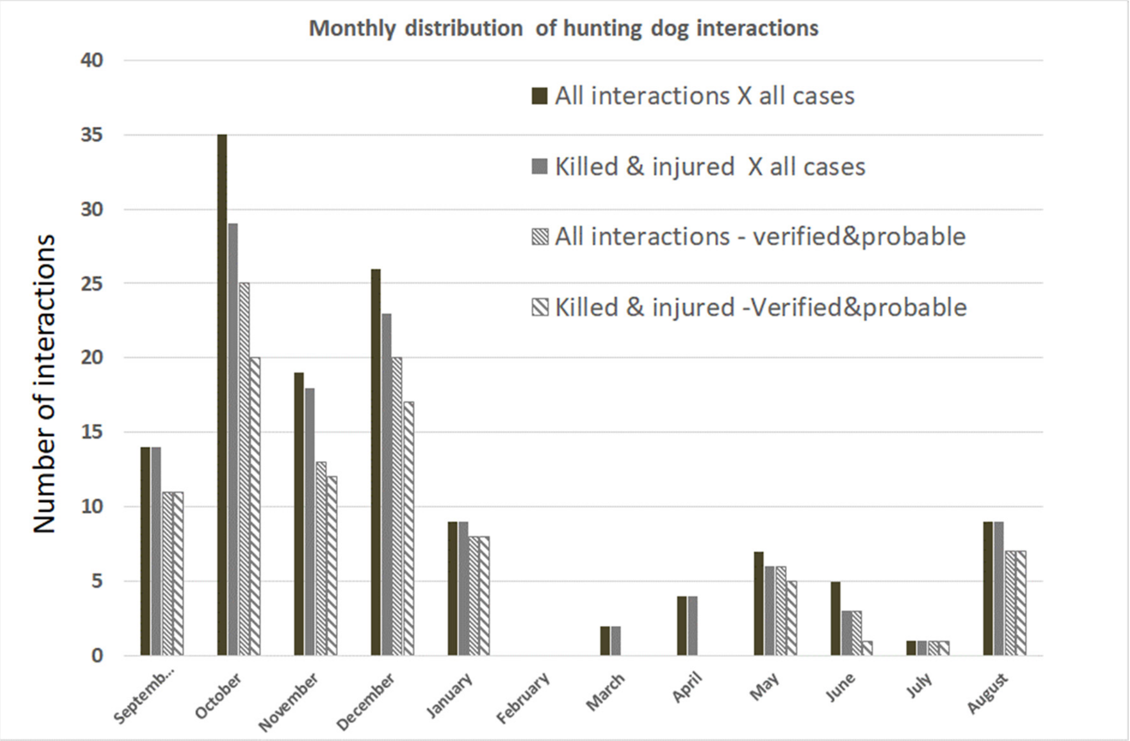

Figure S2. Monthly distribution of wolf-hunting dog interactions in the study area according to interaction and verification categories.

Figure S3

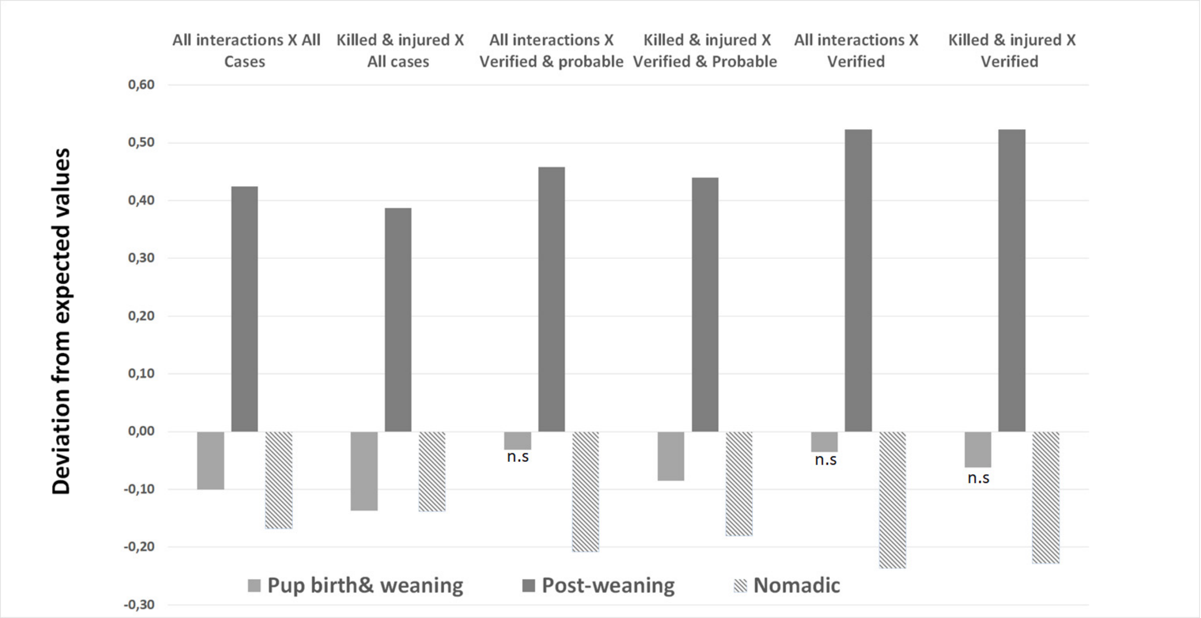

Figure S3. Deviations from expected frequency distributions of hunting dog interactions among wolf seasons for all available data sets. All deviations are significant at $p<0.01$ expect when those are denoted as "n.s" under the deviation bars.

Figure S4

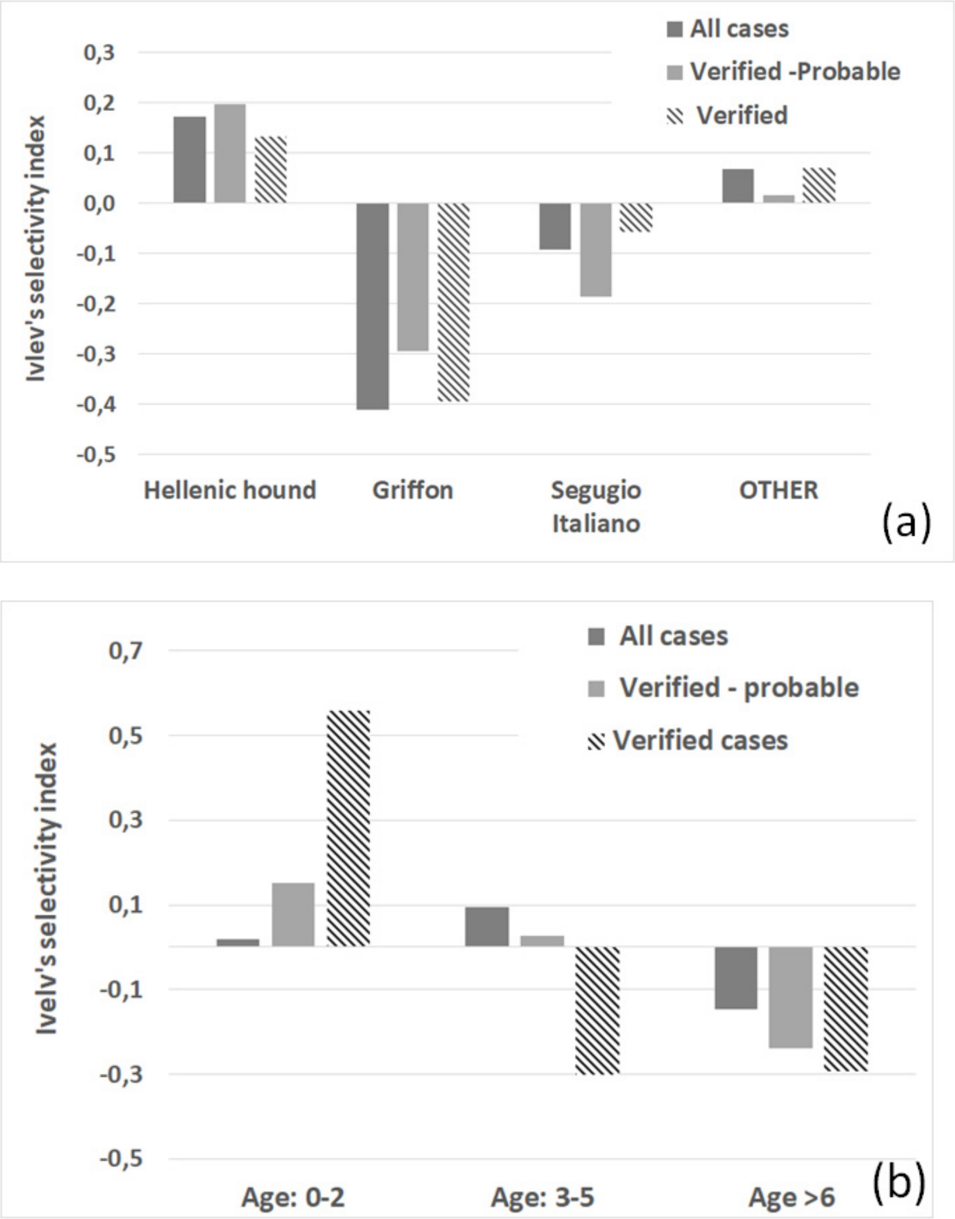

Figure S4. Selection of (a) dog breeds or groups and (b) age classes according to their susceptibility to mortality and injury for the three verification data sets.

Figure S5

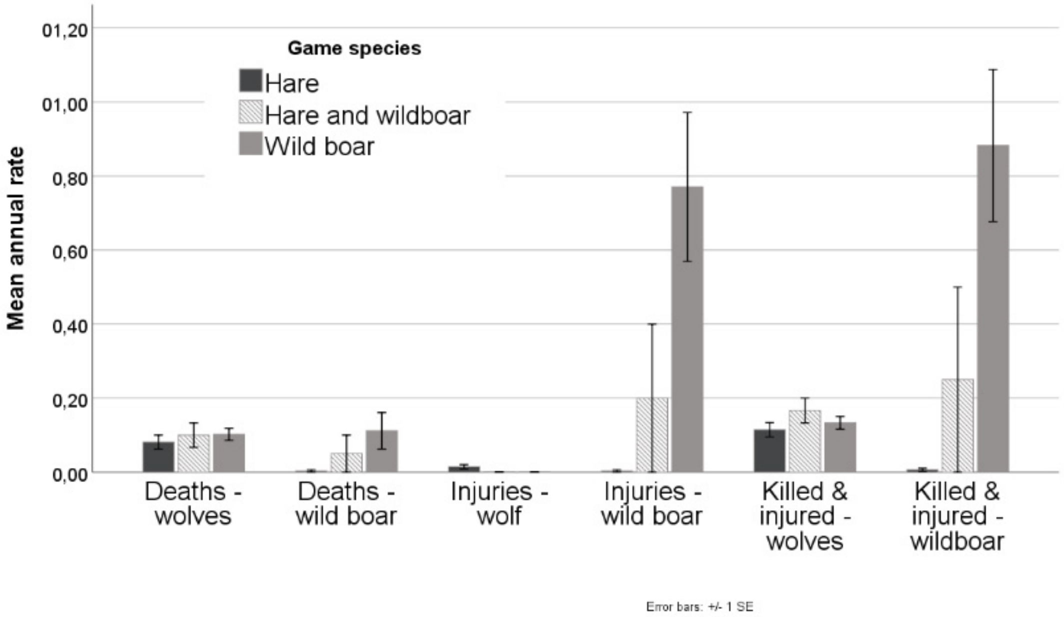

Figure S5. Mean annual rates of dog injuries and fatalities per species involved (wolf or wild boar) for each hunter/game category. For wolf-dog interactions the "verified& probable" data set was used. Error bars represent standard errors of the mean.

Figure S6

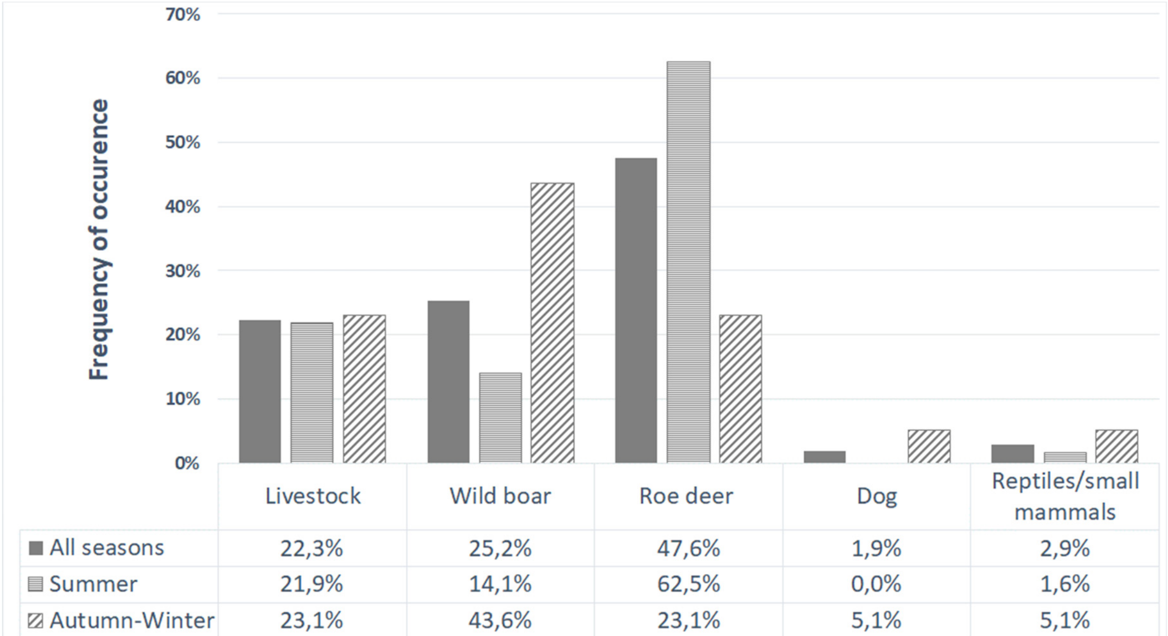

	Livestock	Wild boar	Roe deer	Dog	Reptiles/small mammals
All seasons	22,3%	25,2%	47,6%	1,9%	2,9%
Summer	21,9%	14,1%	62,5%	0,0%	1,6%
Autumn-Winter	23,1%	43,6%	23,1%	5,1%	5,1%

Figure S6. Seasonal frequency of occurrence (FO%) of prey items identified in the wolf scat sample from the study area (n=103)

Table S3. Replicated bootstrapped models of dog predation risk in the study area. Models are ranked according to their average performance in all 4 criteria of model transferability (AUC*test*, AUC*diff*, OR10 and OR*min*). Low values of the average ranking score indicate less overfitted and with better transferability models.

Model rank	**AUCtrain**	**AUCtest**	**AUCdiff**	**10% training presence test omission (OR10)**	**Minimum training presence test omission (ORmin)**	**Average Ranking Score**
1	0,8235	0,7363	0,087	0,1821	0,0318	3
2	0,8212	0,7331	0,088	0,1891	0,0353	5
3	0,8043	0,7204	0,084	0,1946	0,0332	7
4	0,8055	0,7219	0,084	0,1856	0,0443	7
5	0,8152	0,7252	0,090	0,1932	0,0395	8
6	0,8385	0,7565	0,082	0,2091	0,0457	9
7	0,8084	0,7198	0,089	0,1939	0,0415	9
8	0,8231	0,7393	0,084	0,1967	0,0665	10
9	0,8176	0,7270	0,091	0,1974	0,0457	11
10	0,8347	0,7377	0,097	0,2064	0,0429	12
11	0,8009	0,7116	0,089	0,2015	0,0353	12
12	0,8086	0,7138	0,095	0,1960	0,0429	13
13	0,7947	0,7147	0,080	0,2036	0,0478	13
14	0,8209	0,7207	0,100	0,2098	0,0381	14
15	0,8334	0,7392	0,094	0,2195	0,0499	15
16	0,8126	0,7175	0,095	0,1988	0,0457	15
17	0,8171	0,7205	0,097	0,2174	0,0367	15
18	0,8189	0,7273	0,092	0,2133	0,0540	16
19	0,8140	0,7185	0,095	0,2008	0,0464	16
20	0,8279	0,7276	0,100	0,2119	0,0512	17
21	0,8137	0,7179	0,096	0,2126	0,0457	18
22	0,8089	0,7094	0,099	0,2140	0,0436	19
23	0,8092	0,7069	0,102	0,2154	0,0429	20
24	0,8277	0,7208	0,107	0,2230	0,0547	21
25	0,8139	0,7092	0,105	0,2313	0,0519	24

Figure S7

a

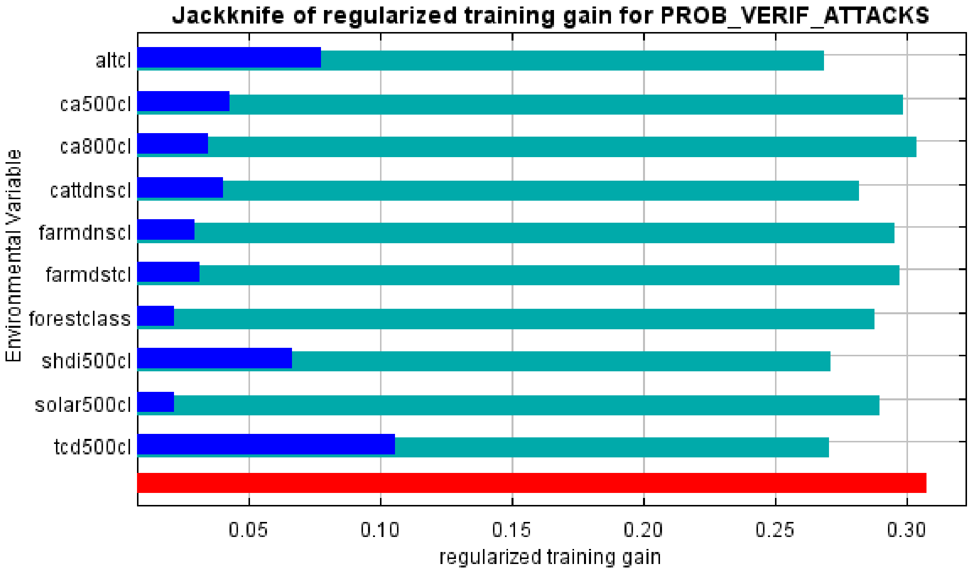

b

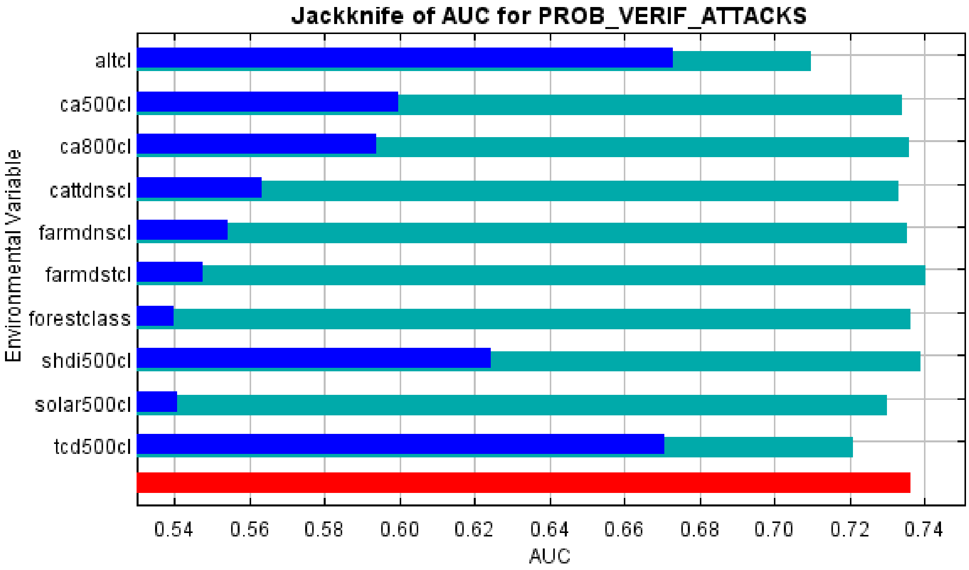

Figure S7. **(a)** Jackknife tests of the training (upper graph) and **(b)** AUC gain (lower graph) in the highest ranked model. The bars indicate the explanatory power when only the specific variable is included in the MaxEnt model (dark blue) and the explanatory power of the full model when the variable is omitted (light blue). **Altcl**= Altitude, **CA500cl** = Forest Core area at 500m radius, **CA800cl** = Forest Core area at 800m radius**, Cattlednscl=** Cattle density in head/km2, **Farmdnscl**= Livestock farm density in farms/ km2, **Farmdstcl**= Distance from livestock farms in meters, **Forest class** = basic forest class categories, **Shdi 500cl** = Shannon diversity index at 500m radius, Solar **500cl=** Yearly solar radiation at a500m, **TCD500cl** = Tree Cover Density at 500m radius. Parameters in Jackknife graphs appear in alphabetical order.

Figure S8

a

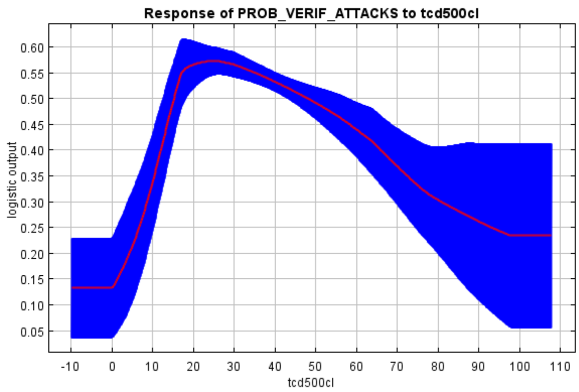

b

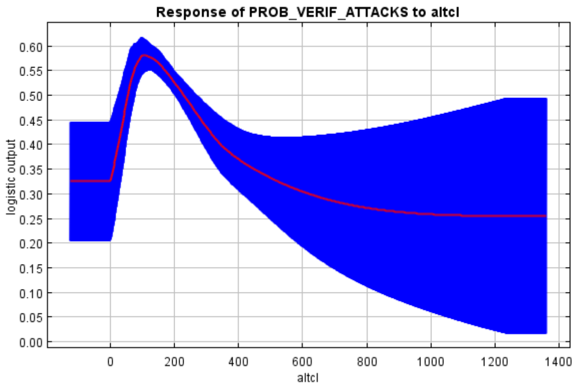

c

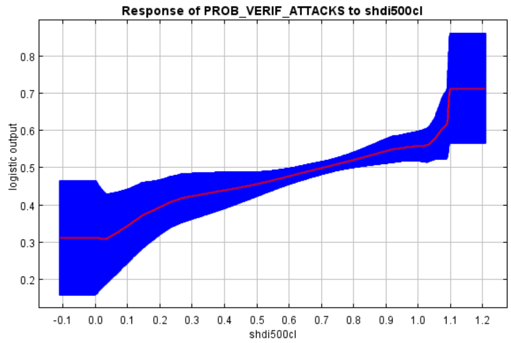

d

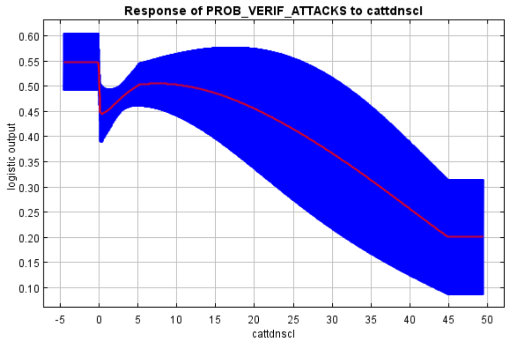

e

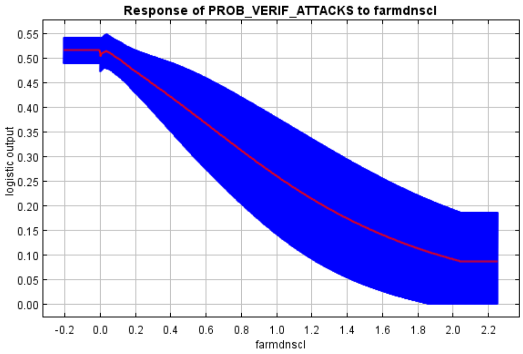

f

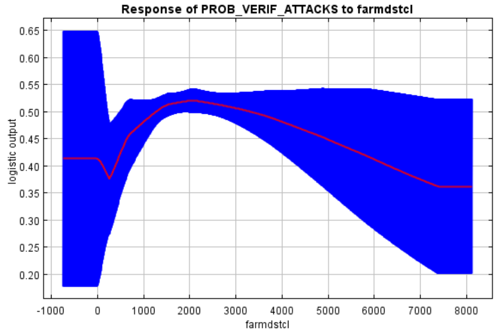

g

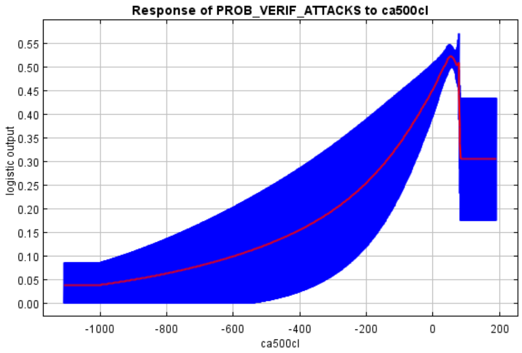

h

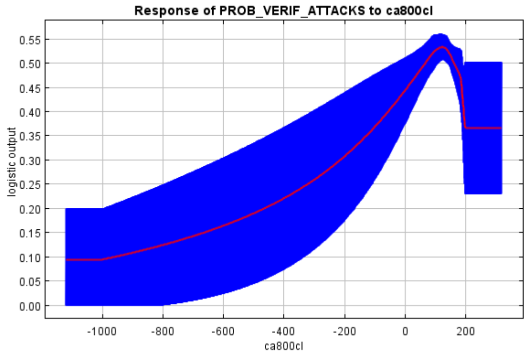

i

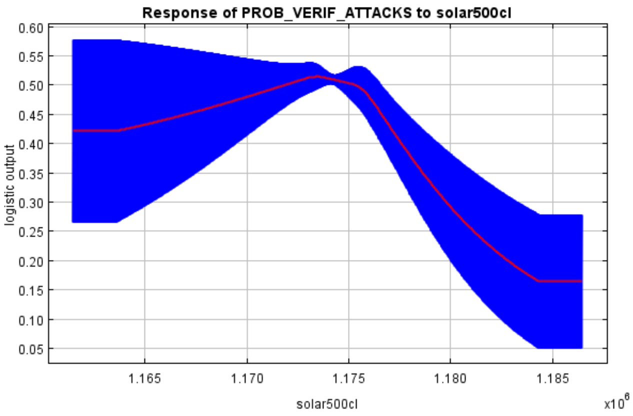

j

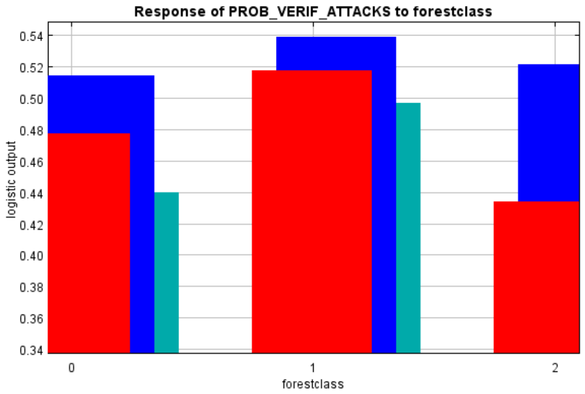

Figure S8. Averaged response curves (red line) for variables retained in the bootstrapped replicated best Maxent model, with respective confidence intervals (blue areas): a) "*Tree cover density at 500m*" b) "*Altitude*" c) "*Shannon diversity index*", d) "*Cattle density*" e) "*Livestock Farm density*" f) "*Distance from livestock farms*" g) "*Forest core area at 500m*" h) "*Forest core area at 800m*" i) "*Yearly solar radiation*" j) "*Forest Class*". (0=no forest, 1= Broadleaved/scrublands, 2= Conifers)

Table S4

Table S4. Percent distribution and permutation importance of variables used in Maxent best replicated model related to dog-predation risk. Variables are presented in a descending order according to their percent contribution.

Variable	Percent contribution	Permutation importance
**Tree cover density at 500m**	20.4	21.9
**Altitude**	18.3	19.4
**Shannon diversity index at 500m radius**	14.2	9.1
**Cattle density**	11.2	8.9
**Forest Class**	8.6	6.9
**Forest core area at 500m**	7.7	7.5
**Yearly solar radiation at 500m radius.**	6	6.7
**Livestock farm density**	6	12.5
**Distance from livestock farms**	4.2	3.4
**Forest core area at 800m**	3.5	3.8

Figure S9

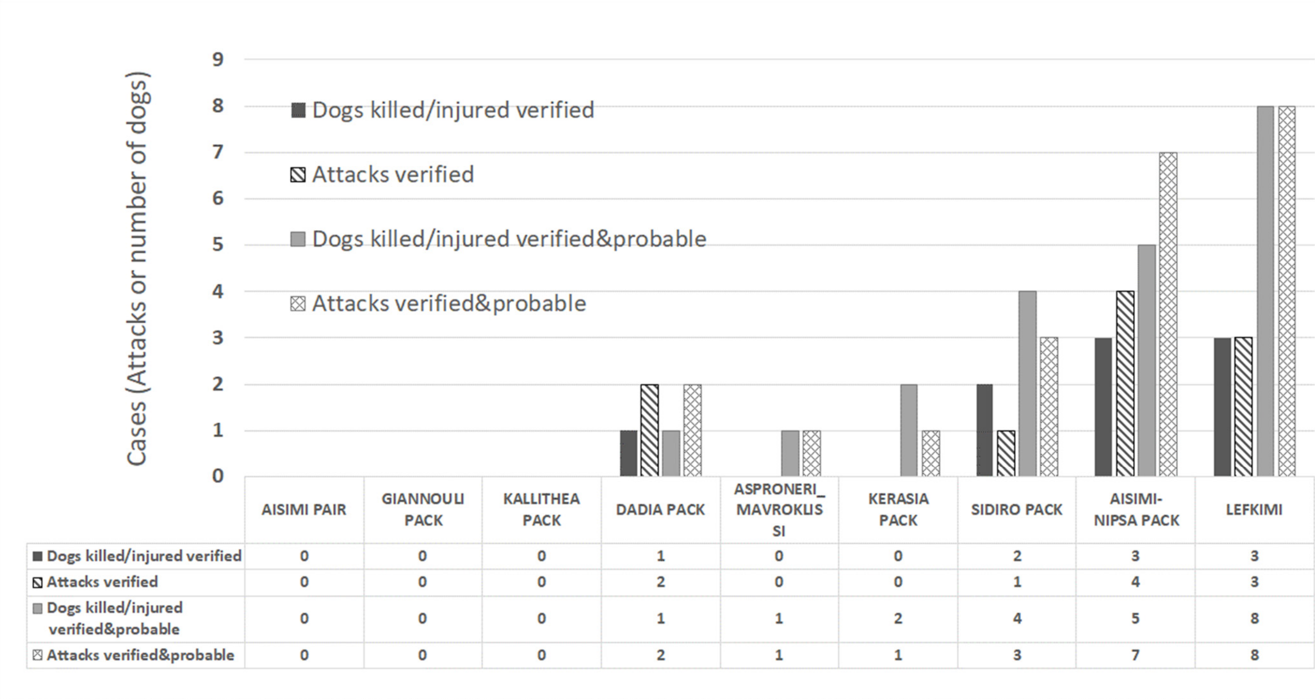

	AISIMI PAIR	GIANNOULI PACK	KALLITHEA PACK	DADIA PACK	ASPRONERI_ MAVROKLISSI	KERASIA PACK	SIDIRO PACK	AISIMI-NIPSA PACK	LEFKIMI
Dogs killed/injured verified	0	0	0	1	0	0	2	3	3
Attacks verified	0	0	0	2	0	0	1	4	3
Dogs killed/injured verified&probable	0	0	0	1	1	2	4	5	8
Attacks verified&probable	0	0	0	2	1	1	3	7	8

Figure S9. Number of "verified" and "verified & probable" dog interactions and events per wolf social unit in the project area during 2019 and early 2020.

Reference list

1. Copernicus Land Monitoring Service Available online: https://land.copernicus.eu/ (accessed on Aug 30, 2021).
2. McGarigal, K.; Cushman, S.A.; Ene, E. FRAGSTATS v4: Spatial Pattern Analysis Program for Categorical and Continuous Maps. Computer software program produced by the authors at the University of Massachusetts, Amherst, USA 2012.
3. Hellenic National Statistical, A. *Data on livestock availability per local community in the project area for the year 2012*;
